# *Operando* Contactless EFISH Study of the Rate-Determining Step of Light-Driven Water Oxidation on TiO_2_ Photoanodes

**DOI:** 10.1021/jacs.5c01836

**Published:** 2025-05-21

**Authors:** Fengyi Zhao, Zihao Xu, Sa Suo, Yixuan Xu, Craig L. Hill, Djamaladdin G. Musaev, Tianquan Lian

**Affiliations:** † Department of Chemistry, 1371 Emory University, Atlanta, Georgia 30322, United States; ‡ Cherry L. Emerson Centre for Scientific Computation, Emory University, 1515 Dickey Drive, Atlanta, Georgia 30322, United States

## Abstract

For many slow solar-fuel-forming reactions, the accumulation of photogenerated minority carriers on the photoelectrode surface leads to light-induced band edge unpinning, affecting the junction properties by decreasing band bending in the semiconductor space charge layer and increasing the driving force of surface reactions in the electric double layer. In this study, we demonstrate a contactless *operando* electric field-induced second harmonic generation (EFISH) method for measuring the band bending change (δΔΦ_SCR_^L^) on photoelectrodes upon photoexcitation. For n-doped rutile TiO_2_ water oxidation photoanodes at pH 7, δΔΦ_SCR_^L^ increases at more positive potentials or higher illumination power density until it reaches saturation values. We show that under fast mass transport conditions, δΔΦ_SCR_^L^ is exclusively attributed to the accumulated charged rate-determining species that can be regarded as temporary surface states, and the relationship between the photocurrent and δΔΦ_SCR_^L^ can be well modeled by assuming that hole trap states function as the reaction center. Kinetic isotope experiments identify proton-coupled electron transfer as the rate-determining step and suggest a possible chemical nature of the key intermediate. We demonstrate that light-induced band edge unpinning is a beneficial feature under high illumination conditions for oxygen evolution reaction on TiO_2_ because it maintains the photon-to-current conversion efficiency by enhancing the surface reaction driving force, shedding light on the actual device application.

## Graphical abstract

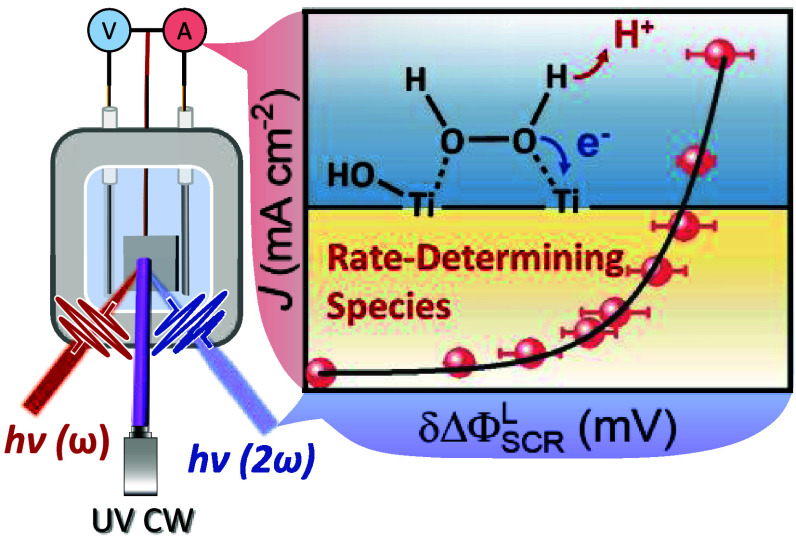

## Introduction

The electrostatic potential profile across the semiconductor/electrolyte junction plays an essential role in efficient photoelectrochemical processes that store solar energy as chemical fuels. On the semiconductor side and under low-level injection conditions, the potential difference (i.e., band bending, ΔΦ_SCR_) across the space charge region (SCR) drives the photogenerated minority carriers to the surface and majority carriers to the bulk, maintaining the quasi-Fermi level of holes that drive the reaction.^1–3^ On the electrolyte side, the potential drop across the electric double layer (EDL), ΔΦ_EDL_, influences the surface charge carrier energetics and their reaction kinetics, as illustrated in Figure 1a.^4–7^ In kinetically sluggish solar-fuel-forming reactions on semiconductor photoelectrodes, such as water oxidation or CO_2_ reduction, the photogenerated minority carriers can accumulate on the surface, altering the electrostatic potential distribution across the semiconductor/electrolyte junction (Figure 1b). This results in reduced band bending (δΔΦ_SCR_) in semiconductor and increased electrostatic potential drop (δΔΦ_EDL_) in the EDL, as shown in Figure 1b, depicting band diagrams under a specific 1 V vs. Ag/AgCl applied potential. This phenomenon, termed “light-induced Fermi level pinning”, “band flattening”, or “light-induced band edge unpinning”,^1,2,8–10^ affects several key junction properties such as charge separation, surface recombination, and reaction driving force, significantly influencing the device performance. Despite many reports of light-induced band edge unpinning, the chemical origin of surface charge and the effect of light-induced band edge unpinning on junction properties and photoelectrochemical performance are still questions of considerable debates.^7,11–19^ For example, it has been suggested that surface states causing light-induced band edge unpinning function both as the recombination center and reaction site for charge transfer to redox couples;^11–13^ surface states originated from ion adsorption from the solution can act as faradaic mediators;^14^ the increased δΔΦ_EDL_ from surface minority carrier accumulation increases the driving force and rate constant for redox reactions.^7,15–18^ In some cases, models with and without consideration of light-induced band edge unpinning led to very different reaction mechanisms.^19,20^

**Figure 1:**
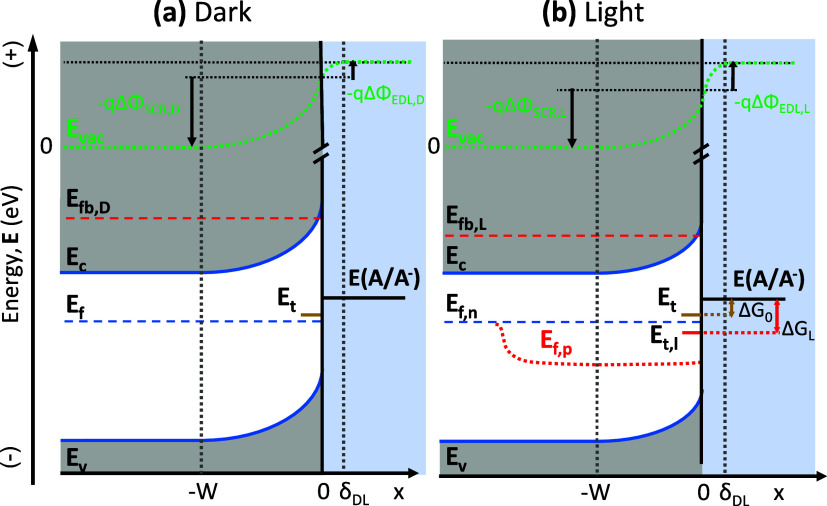
Illustration of energetics alignment of n-semiconductor–electrolyte junction in the depletion region (under 1 V vs. Ag/AgCl applied potential) under (a) dark and (b) light illumination conditions. Color codes: local vacuum energy level (**E_vac_**, unit in eV, green dotted line), conduction (**E_c_**) and valence (**E_v_**) band edge energy level (unit in eV, blue solid lines), Fermi level of electron (**E_f_**, blue dashed lines), quasi-Fermi level of electrons and holes, respectively (**E_f,n_**, **E_f,p_**, unit in eV, red dotted curve), Fermi level under flatband conditions in the dark and light (*E*_fb,D_, *E*_fb,L_, unit in eV, red dashed lines), trap state energy with (**E_t,l_**) and without (**E_t_**) light-induced band edge unpinning (unit in eV), and solution redox energy level (**E­(A/A^–^)**, unit in eV). Also shown are the depletion width (W) in the SCR, the width of the electric double layer in the electrolyte (δ_DL_), the total electrostatic potential variation in the SCR (ΔΦ_SCR,i_, unit in V), and in the electric double layer (ΔΦ_EDL,i_, unit in V) in the dark (i = D) and under illumination (i = L), and the driving force for reaction for the trapped hole with (ΔG_L_) and without (ΔG_0_) light-induced band edge unpinning (unit in eV). Accumulation of photogenerated holes on the surface-trapped states increases the electrostatic potential different from the solution to the electrode surface, which lowers the energy of states at the surface (**E_c_**, **E_v_**, and **E_t_**) and increases the driving force for hole transfer reaction to the redox couple.

A major challenge in studying the effect of light-induced band edge unpinning is the lack of *in situ* or *operando* tools for directly measuring the band edge unpinning in semiconductor photoelectrodes. While the total electrostatic potential difference between the bulk of the semiconductor and electrolyte solution depends on the applied electrode potential (U_app_), the partition between the potential drop in SCR and EDL is governed by their capacitances, C_SCR_ and C_EDL_, respectively. Early studies on light-induced band edge unpinning mainly utilized electrochemical methods, including electrochemical impedance spectroscopy,^5,15,17^ intensity modulated photocurrent spectroscopy,^18,21,22^ and photocurrent analysis.^11–13,23^ Relating these electrochemical measurement results to the change of semiconductor band bending can be difficult for complex and/or nanostructured semiconductor/electrolyte junctions.^24^ Recently, several new techniques have been developed to investigate electrostatic potential profiles at the (photo)­catalytic interface. For instance, a dual-working electrode was developed for *in situ* probing of the surface potential of a semiconductor electrode or a cocatalyst.^25–27^ However, this method typically mandates an ion-permeable ohmic contact between the working electrode and the probe electrodes, which may alter the surface properties. A more recent development employs potential-sensing electrochemical atomic force microscopy to map the surface voltages in nanoscale photocatalytic structures.^28–30^ Nonetheless, the method requires close contact and conductivity between the AFM tip and target interface to achieve quasi-Fermi level equilibrium. These contact-based methods, however, are not applicable to semiconductor macro-crystal photocatalysts, where band bending also plays an important role.^31,32^ Noncontact optical methods, such as sum-frequency generation^33,34^ and Raman spectroscopy,^4,35–37^ have been used to characterize electric field changes in the EDL by measuring the vibrational frequency shifts of surface-bound molecules or catalysts. However, these methods often require adsorbed molecular Stark effect probes, which may alter the junction properties, particularly those related to surface trap states.^38,39^ Thus, there is a need for developing contactless *in situ*/*operando* methods for measuring electrostatic potential profiles at photoelectrode/electrolyte interfaces without surface modification.

The electric field-induced second harmonic generation (EFISH) technique provides a general contactless approach to characterize electric fields and electrostatic potential at electrode/electrolyte junctions.^9,10,40–43^ Additionally, EFISH can achieve high time resolution to monitor photogenerated carrier dynamics.^10^ In our previous work, we demonstrated a contactless EFISH method to characterize the electrostatic potential profile of an n-type rutile (100) TiO_2_/electrolyte junction under dark conditions, covering potentials from semiconductor depletion to accumulation regions and quantifying the electrostatic potential profiles in the SCR and EDL.^43^ In this study, we apply the EFISH method on the same n-TiO_2_/electrolyte interface to study the more important light-driven water oxidation reaction in an *operando* manner, under which conditions are not accessible under the previous study. Band bending changes under illumination due to surface hole accumulation at midgap states. The relationship between the photoanode band bending changes and water oxidation photocurrent can be well modeled by assuming pseudo-first-order reaction kinetics of accumulated holes in the surface state. The chemical nature of surface-accumulated holes is investigated via the isotope effect on photocurrent and band bending change, which shows that the oxygen evolution reaction (OER) rate-determining step on the TiO_2_ photoanode involves a proton-coupled electron transfer (PCET) reaction, aligning with the previously reported surface peroxo OER rate-determining species. While light-induced band edge unpinning leads to a positive shift of photocurrent onset potential, it maintains the absorbed photon-to-current conversion efficiency (APCE) at high illumination conditions by increasing the interfacial reaction rate constant.

## Results and Discussion

### *Operando* EFISH on TiO_2_ under Illumination

The second harmonic generation (SHG) signal intensity, I­(2ω), at the semiconductor/electrolyte interface is described by eq 1.^43^

1 $$I\left(2\omega \right)\propto {\left|\chi_{\mathrm{eff}}^{\left(2\right)}+\chi_{\mathrm{SCR}}^{\left(3\right)}{\Delta \Phi }_{\mathrm{SCR}}+\chi_{\mathrm{EDL}}^{\left(3\right)}{\Delta \Phi }_{\mathrm{EDL}}\right|}^{2}{I\left(\omega \right)}^{2}$$

In eq 1, χ_eff_^(2)^ is the effective second-order susceptibility of the solid surface and oriented interfacial electrolyte layers; χ_SCR_^(3)^ and χ_EDL_^(3)^ correspond to the effective third-order susceptibilities at the semiconductor SCR and solution EDL, respectively; ΔΦ_SCR_ = Φ­(−W) – Φ(0) denotes the electrostatic potential difference (band bending) between the bulk and surface of the semiconductor, occurring over the SCR, and ΔΦ_EDL_ = Φ(0) – Φ­(δ_DL_) is the electrostatic potential difference across the EDL, as shown in Figure 1a. In our previous work,^43^ we referred to these as ΔΦ_sc_ and ΔΦ_liq_ to indicate the potential drop in the semiconductor side and liquid side, respectively, that contribute to the EFISH signal. As discussed in that paper, under our experimental conditions, the signal originates from the whole SCR and EDL, and the notations were used to specify their spatial region.

For n-type photoelectrodes in the depletion region, where the applied electrode potential (U_app_) is more positive than the flatband potential in the dark (U_fb_^D^), most of the junction electrostatic potential change (ΔΦ) resides in the SCR due to its much smaller capacitance compared to the EDL, as depicted in Figure 1a. Hence, in the dark, ΔΦ_SCR_^D^ ∼ (U_app_ – U_fb_^D^), while the electrostatic potential drop across the EDL can be considered to be bias-independent and equal to its value at the flatband condition, ΔΦ_EDL_^D^ ∼ ΔΦ_EDL_^fb^.^44,45^ The SHG intensity of the junction in the depletion region under dark conditions can be expressed as

2 $${I\left(2\omega \right)}^{D}=C{\left|\left|\chi^{\mathrm{fb}}\right|+e^{i\alpha }\left|\chi_{\mathrm{sc}}^{\left(3\right)}\right|\left(U_{\mathrm{app}}-U_{\mathrm{fb}}^{D}\right)\right|}^{2}{I\left(\omega \right)}^{2}$$

In eq 2, χ^fb^ = χ_eff_^(2)^ + χ_EDL_^(3)^ΔΦ_EDL_^fb^ represents the effective susceptibility at the flatband potential (denoted as χ_bid_ in our previous paper^43^), α is the phase difference between χ_SCR_^(3)^ and χ^fb^, and *C* is a proportionality constant. This relationship was confirmed in our recent EFISH study on n-type rutile (100) TiO_2_/electrolyte junctions.^43^

Under light illumination during slow catalytic reactions, photogenerated minority carriers (holes for n-doped photoelectrodes) accumulate on the surface. Lacking charge compensation will lead to a change in potential distribution across semiconductor/electrolyte junctions (Figure 1b). Specifically, upon illumination, band bending in SCR changes by δΔΦ_SCR_^L^ = ΔΦ_SCR_^D^ – ΔΦ_SCR_^L^ ≈ (U_app_ – U_fb_^D^) – ΔΦ_SCR_^L^, and electrostatic potential drop in the EDL changes by δΔΦ_EDL_^L^ = ΔΦ_EDL_^D^ – ΔΦ_EDL_^L^. Since the potential drop across the bulk semiconductor and bulk solution is kept constant, the amount of band bending decrease in SCR equals the amount of electrostatic potential drop increases in EDL. We have δΔΦ_SCR_^L^ = −δΔΦ_EDL_^L^. The SHG intensity under illumination is expressed as

3 $${I\left(2\omega \right)}^{L}={C\left|\left|\chi^{\mathrm{fb}}\right|+e^{i\beta }\left|\chi_{\mathrm{EDL}}^{\left(3\right)}\right|{\delta \Delta \Phi }_{\mathrm{SCR}}^{L}+e^{i\alpha }\left|\chi_{\mathrm{sc}}^{\left(3\right)}\right|\left[{(U}_{\mathrm{app}}-U_{\mathrm{fb}}^{D}\right)-{\delta \Delta \Phi }_{\mathrm{SCR}}^{L}]\right|}^{2}{I\left(\omega \right)}^{2}$$

In eq 3, β denotes the relative phase difference between χ_EDL_^(3)^ and the bias-independent term χ^fb^. It should be noted that not only the accumulated surface-trapped holes but also surface adsorption of any kind of positive ionic charges derived from the photoelectrochemical process can lead to light-induced band edge unpinning, which can also be described by eq 3.

Figure 2a illustrates the experimental setup for an *operando* EFISH study on an n-type rutile TiO_2_(100)/electrolyte junction. The 800 nm fundamental pulses and 360 nm continuous wave (CW) illumination beam for TiO_2_ excitation were applied at 45 and 0° incident angles, respectively. The TiO_2_[001] axis was aligned parallel to the incidental plane, with fundamental and SHG beams s- and p-polarized, respectively. Our prior crystal azimuthal angle and light polarization dependence study demonstrated that under the above-mentioned conditions, the bias-dependent SHG intensity in the dark can be well described by eq 2 with α ∼ 90°, resulting in a signal minimum at the TiO_2_ flatband potential U_fb_^D^.^43^ Under these conditions, the SCR EFISH signal in the depletion potential region is significantly larger than the EDL, and the second term in eq 3 can be neglected. EFISH experiments were performed in 1 M NaClO_4_ solution under varying light illumination intensities during the negative scan of cyclic voltammetry (CV), as shown in Figure 2b. The electrolyte solution was stirred during measurement to allow for fast mass transport to prevent surface pH variations. EFISH measurements can be treated as steady-state readings at specific potentials due to the slow potential scan rate (3.53 mV/s). All potentials in this paper are referenced to Ag/AgCl (1 M KCl) unless stated otherwise.

**Figure 2:**
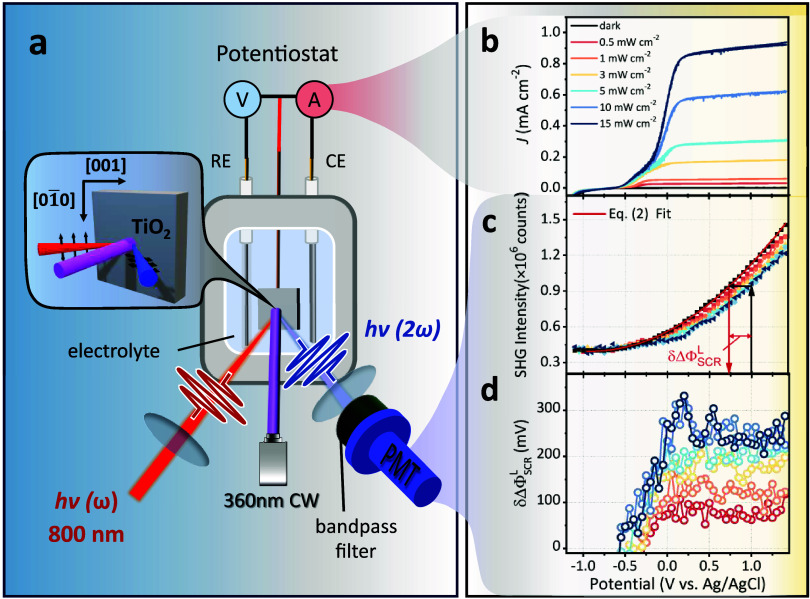
(a) Schematic illustration of a steady-state EFISH setup. The zoom-in panel indicates the adopted TiO_2_ azimuthal and light polarization conditions of measurements. 1 kHz 35 fs 800 nm pulses are used as the fundamental light for the EFISH measurement, and continuous wave (CW) 360 nm light-emitting diode (LED) light is used as the UV illumination source. (b) Current densities as a function of applied potential at indicated illumination power densities measured by CV with a scan rate of 3.53 mV/s. (c) EFISH intensity as a function of applied potential on rutile TiO_2_ (100) under 360 nm UV illumination with different power densities. Also shown is an example of determining the δΔΦ_SCR_^L^ value at 1 V under 15 mW/cm^2^ illumination power density using eq 3. (d) Change in semiconductor band bending δΔΦ_SCR_^L^ as a function of applied potential. All of the above experiments were done in 1 M NaClO_4_ electrolyte under stirring conditions. EFISH was measured under a negative scan direction.

Figure 2b shows the typical current response under varying illumination intensities on TiO_2_ photoanode: photocurrent increases with U_app_ starting from −0.5 V and saturates after a certain positive potential under the same illumination intensity; saturated photocurrent densities increase with light intensity in a roughly linear fashion. It should be noted that a small photocurrent plateau is observed at ca. −0.25 V, especially at high illumination intensity. This feature is only observed at pH 7 unbuffered solution under fast mass transport conditions. One possible explanation is that the first phase of photocurrent growth corresponds to the electron–hole recombination current that relates to surface states, while the second phase of the photocurrent growth corresponds to the photooxidation reaction.^46^ In our later analysis, we report the photocurrent onset potential only based on the second phase of photocurrent growth. Figure 2c shows that under dark conditions, EFISH intensities in the semiconductor depletion region exhibit a quadratic response to U_app_ and can be well fit by eq 2 with a minimum at U_fb_^D^ = −0.933 ± 0.019 V. This agrees well with the U_fb_^D^ value obtained via the Mott–Schottky method (Figure S3d). The fit also reveals the value of C|χ^fb^| and C|χ_SCR_^(3)^|, which can be used to calculate δΔΦ_SCR_^L^ under light illumination conditions according to eq 3. Upon 360 nm CW excitation, EFISH intensities decrease from their dark response levels starting from ca. −0.5 V to more positive potentials. According to eq 3, this indicates reduced band bending in the SCR, which is shown in Figure 2d as a function of the applied potential. The result shows that δΔΦ_SCR_^L^ increases with applied potential under a fixed illumination power density, plateauing at higher potentials, similar to the photocurrent trends in Figure 2b; δΔΦ_SCR_^L^ values in the plateau region increase at higher illumination power density and saturates at around 10 mW/cm^2^. Although Figure 2c only presents the δΔΦ_SCR_^L^-U_app_ plot acquired under the negative scan direction, data from both scan directions are highly repeatable with negligible hysteresis (Figure S2). What needs to be noted is that at high illumination intensities (10 and 15 mW/cm^2^), the “peak” at approximately +0.2 V stems from the increasing measurement error rather than the real reduction of surface charge densities. The amount of accumulated surface charge should saturate upon large bias as both surface recombination and charge transfer rate will become insensitive to increasing U_app_ at large band bending (U_app_ > 0.2 V). The δΔΦ_SCR_^L^ values in the plateau region (U_app_ = 1 V) measured by EFISH are compared with those determined by Mott–Schottky analysis in Figure S3. An excellent agreement was observed between these methods, confirming that EFISH directly measures the change of band bending upon illumination arising from the photoinduced surface positive charge accumulation.

The unbuffered pH 7 solution used in this study may generate a surface proton gradient during the photoinduced OER process. It is necessary to determine whether our stirring conditions mitigate photoinduced local pH gradient changes, which is another important factor that could lead to δΔΦ_SCR_^L^. Figures S4b and S5b show that δΔΦ_SCR_^L^–U_app_ under unstirred conditions exhibits a similar trend but with an earlier onset potential at ca. −0.75 V compared to stirred conditions. At the same positive U_app_, δΔΦ_SCR_^L^ reaches a larger saturation value under unstirred conditions (Figure S5c). This suggests the adsorption of photogenerated positive ionic species, i.e., H^+^, contribute to δΔΦ_SCR_^L^ under slow mass transport.^47,48^ Electrochemical measurements also reveal local proton concentration gradients under unstirred conditions. Cyclic voltammetry shows photoinduced “redox peaks” before the photocurrent onset region under illumination (Figure S4c), while only reductive features appear in the dark. Impedance spectroscopy reveals a large photoinduced surface charge capacitance (C_ss‑no stir_) under unstirred conditions (Figure S4e, details in Note S2), which response potential corresponds to the reduction peak potential of photogenerated species (−0.75 V). These results together indicate that under slow mass transport during the photoelectrochemical water oxidation process, surface protons accumulate during positive CV scans in the semiconductor depletion potential range and get reduced during negative scans in the semiconductor accumulation potential range. Comparing electrochemical results between stirred and unstirred conditions reveals several substantial differences: (i) under stirring, photocurrent onset and plateau potential align with those in buffered pH 7 electrolyte (Figure S5a); (ii) the large photoinduced surface charge capacitance C_ss‑no stir_ at ca. −0.75 V disappears under stirring (Figures S3c,f and S4e); (iii) accelerated mass transport eliminates the photoinduced redox feature in CV, greatly reducing hysteresis between different scan directions (Figure S5d,e). Collectively, these results confirm that our stirring condition effectively eliminates surface proton gradients between the electrode surface and the bulk solution, allowing us to attribute δΔΦ_SCR_^L^ under rigorous stirring conditions solely to the accumulated photogenerated holes. Our experimental findings align with an estimation of surface proton concentrations (Note S3 and Figure S6) and previous ring-disk electrode and electrolyte electroreflectance study.^49,50^

However, the accumulation of holes on the valence band can also unpin the band edge by forming the inversion layer.^51^ We believe that this is highly unlikely on TiO_2_ photoanode, mainly because C_SCR_ did not exhibit a noticeable increase upon light illumination and increasing applied potential (Figure S3c), meaning that the ionized dopant is still the main contributor to the positive charge density within SCR at large band bending. The presence of hole trap states can be revealed by impedance spectroscopy, where photoinduced capacitance peaks are observed from −0.5 to 0 V potential range (Figure S3f and Table S1). This agrees well with the onset potential for light-induced band edge unpinning (Figure 2d). Therefore, we assign the origin of δΔΦ_SCR_^L^ to be the photogenerated holes trapped at the surface states. This also aligns with the assignment of surface-trapped holes from previous studies on metal oxide photoanode.^52–54^ According to a model for light-induced band edge unpinning on n-doped photoanodes, the positive charge at surface states is given by^8,19,54^

4 $$p_{\mathrm{surf},\mathrm{ss}}=C_{\mathrm{EDL}}{\delta \Delta \Phi }_{\mathrm{SCR}}^{L}/q$$

In eq 4, C_EDL_ is the capacitance of the EDL, often assumed to be dominated by the contribution of the Helmholtz layer, and *p*_surf,ss_ is the surface state charge hole density, which arises from the hole trapping on the surface state. What needs to be mentioned is that to emphasize the quantity of light-induced band edge unpinning, we define δΔΦ_SCR_^L^ in a different way compared to previous work,^8,19^ leading to a positive value describing the band bending changes. Using the measured saturated δΔΦ_SCR_^L^ value from Figure 2d and the estimated value of C_EDL_ = 50 μF/cm^2^,^47^ the surface hole density under 10 mW/cm^2^ illumination power density is calculated to be 7.6 × 10^13^ cm^–2^, which agrees with the value from previous transient photocurrent analysis (ca. 10^13^–10^14^ cm^–2^) on n-TiO_2_,^50,54^ corresponding to about 1.5% of surface atoms being positively charged with a hole.^55^

To gain more insight into the potential dependent δΔΦ_SCR_^L^ behavior, we compare the EFISH method results to classic transient photocurrent measurements. Figure 3a shows the TiO_2_ transient photocurrent obtained via chop-light chronoamperometry. At an intermediate applied potential, an anodic photocurrent spike with current density (*J*_0_^+^) and a cathodic transient current overshoot (*J*_0_^–^) are obtained when turning on and off the illumination, representing the maximum photogenerated hole flux density and the recombination current density, respectively.^1,56^ A steady-state photocurrent density (*J*_inf_) develops when the hole flux density balances charge transfer and recombination rates. In an ideal model where surface-accumulated minority carriers do not alter the semiconductor/electrolyte junction potential distribution, the anodic spike decay (*J*_0_^+^ – *J*_inf_) should equal the value of the cathodic current overshoot (−*J*_0_^–^).^57^ However, our measurements show asymmetry between on and off current spikes, indicating light-induced band edge unpinning.^19^ This asymmetry can be defined using Δ*J*_ph_ = *J*_0_^+^ – *J*_inf_ + *J*_0_^–^, which represents photocurrent reduction associated with the semiconductor band bending changes δΔΦ_SCR_^L^.^54^ Figure 3b shows that under 10 mW/cm^2^ illumination under moderate applied potential, the Δ*J*_ph_ response range matches the δΔΦ_SCR_^L^ onset potential range (−0.5 to 0.2 V), consistent with the model proposed by Salvador et al.^54^ This qualitative comparison demonstrates that the EFISH method not only provides insights similar to classic transient photocurrent measurements but also offers them in a more direct and *operando* manner by directly reporting the amount of band bending change δΔΦ_SCR_^L^.

**Figure 3:**
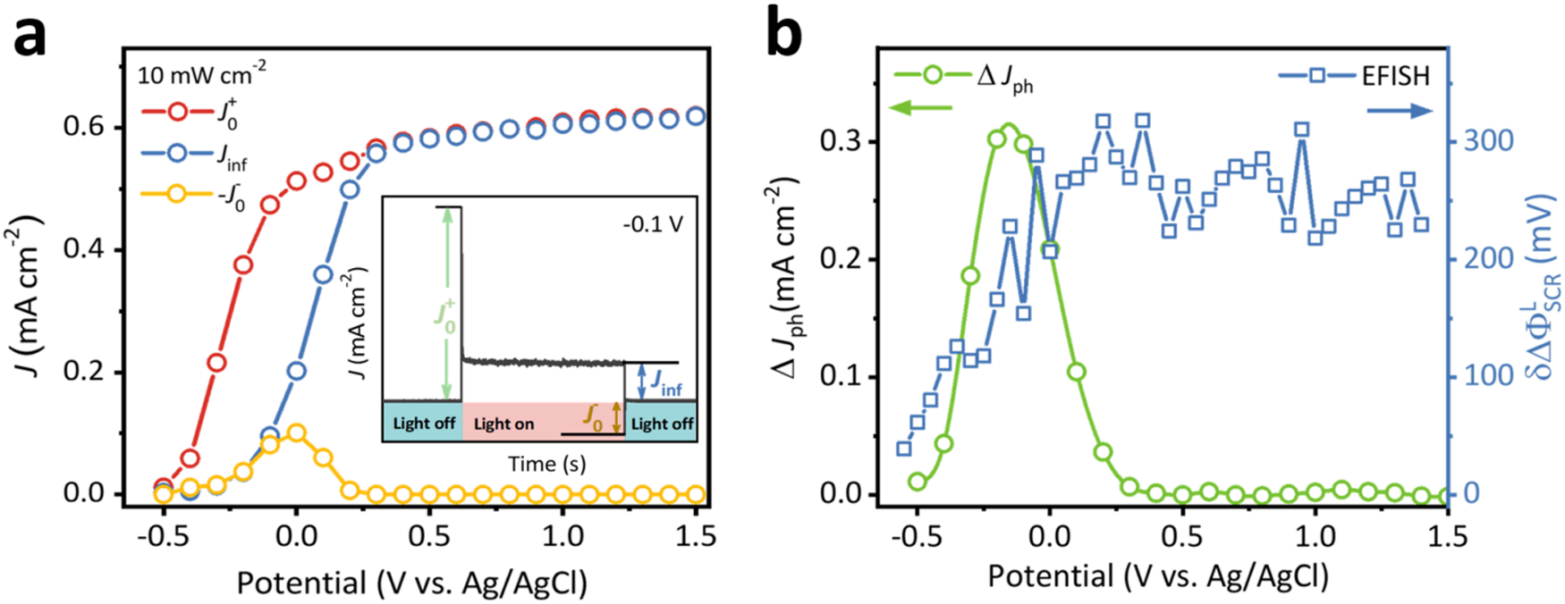
Transient photocurrent measurement on TiO_2_ under 10 mW/cm^2^. (a) Value of anodic spike current density (*J*_0_^+^), steady-state current density (*J*_inf_), and cathodic overshoot current density (−*J*_0_^–^) as a function of applied potential, and the inset illustrates a representative transient current curve obtained from chop-light chronoamperometric measurement at −0.1 V vs. Ag/AgCl and determination of *J*_0_^+^, *J*_inf_, and *J*_0_^–^ values. (b) Δ*J*_ph_ as a function of applied potential, plotted together is δΔΦ_SCR_^L^ under 10 mW/cm^2^ as a function of applied potential. The experiment is measured under rigorous stirring conditions. The lines in (a) and (b) serve as a visual guide.

### Isotope Experiments and Chemical Nature of Rate-Determining Intermediates

Because the *operando* steady-state EFISH measurements are carried out under continuous light illumination and fast mass transport conditions, the trapped holes are associated with the rate-limiting step in photocurrent generation. Following previous literatures, we attribute the surface-trapped holes to the rate-determining reactive intermediates formed by trapping the valence band holes to the surface site.^20,52,58–60^ To investigate the chemical nature of surface-accumulated holes, δΔΦ_SCR_^L^ and corresponding photocurrent are measured in D_2_O, as shown in Figure S7. Both potential dependent δΔΦ_SCR_^L^ and photocurrent exhibit a fashion similar to H_2_O. However, as shown in Figure 4a, δΔΦ_SCR_^L^ in D_2_O exhibits an earlier onset potential and higher values at all potentials compared to H_2_O under 10 mW/cm^2^ power density. The measured δΔΦ_SCR_^L^ values at 1 V U_app_ are also higher in D_2_O than in H_2_O under varying UV illumination power densities (Figure 4b). These results indicate that surface holes accumulate more easily in D_2_O, suggesting a slower hole transfer rate across the TiO_2_/electrolyte junction in D_2_O. Comparison of photocurrent under single illumination intensity (Figure 4c) and illumination-fluence-dependent absorbed photon-to-current efficiency (APCE) (Figure S8, calculation in Note S4) provides insight into photoelectrochemical performance discrepancy between H_2_O and D_2_O. Under 10 mW/cm^2^ illumination power density, D_2_O exhibits a more positive onset potential than H_2_O solution (Figure 4c), suggesting greater surface recombination in D_2_O according to Wilson’s^61^ and Reichman’s^62^ model. Additionally, the OER rate remains lower across the entire photocurrent plateau potential range (U_app_ > 0.2 V) in D_2_O. Figure S8 shows that plateaued APCE decreases with increasing illumination intensity in D_2_O, whereas plateaued APCE in H_2_O remains largely unaffected by higher excitation photon flux. This indicates that in D_2_O, photon conversion efficiency is more susceptible to increased recombination caused by a higher photogenerated carrier concentration. Despite the larger reaction driving force, δΔΦ_SCR_^L^, this vulnerability stems from the smaller intrinsic reaction rate constant and charge transfer coefficient of the rate-determining step, which will be discussed in the subsequent analysis. This result also suggests that the “photocurrent plateau” phenomenon does not necessarily rule out the presence of surface recombination, especially under high photon flux conditions.

**Figure 4:**
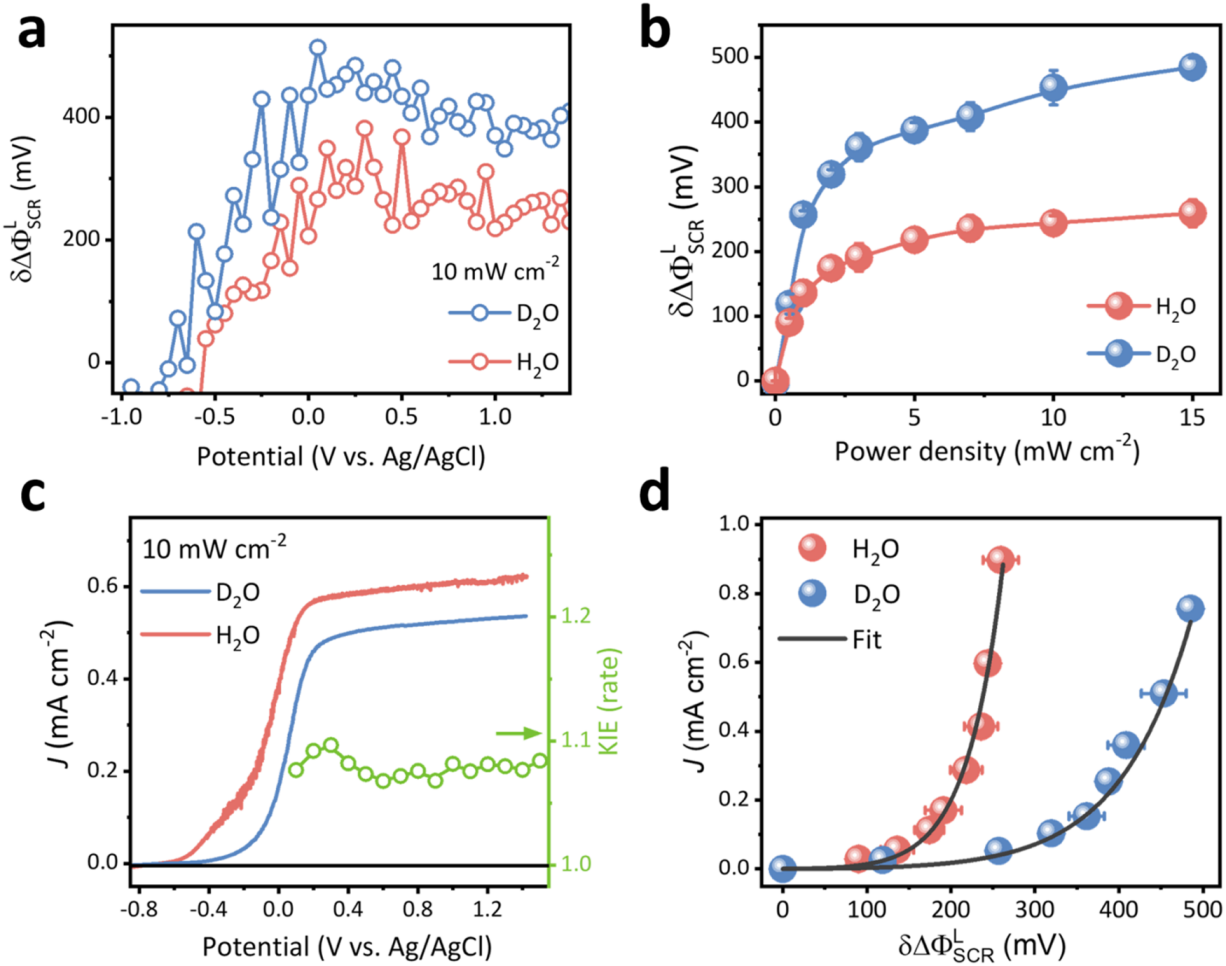
Isotope effect experiment. (a) δΔΦ_SCR_^L^ as a function of applied potential under 10 mW/cm^2^ illumination power density and negative scan direction, (b) δΔΦ_SCR_^L^ as a function of illumination power density measured at 1 V, and (c) photocurrent (left axis) and calculated kinetic isotope effect (KIE) values (right axis) as a function of potential under 10 mW/cm^2^ power density in H_2_O and D_2_O. (d) Photocurrent as a function of corresponding δΔΦ_SCR_^L^ in H_2_O and D_2_O solution at 1 V U_app_ and under varying illumination power densities. The trend is fitted to eq 6. Photocurrent and EFISH data in panels (b) and (d) are collected from a chronoamperometric current measurement under 1 V U_app_. δΔΦ_SCR_^L^ is calculated from EFISH data using eq 3. All of the above experiments used 1 M NaClO_4_ as the electrolyte, H_2_O or D_2_O as the solvent, with solution stirring conditions applied. KIE values are also calculated using the steady-state photocurrent densities measured by chop-light chronoamperometric measurement, as depicted in Figure 3a inset.

These isotopic experimental results indicate that the rate-determining step is sensitive to the proton-coupled electron transfer (PCET) process. The kinetic isotope effect (KIE) can be reported by the steady-state water oxidation rate ratio between H_2_O and D_2_O, i.e., $\mathrm{KIE}\left(\mathrm{rate}\right)=\frac{J(H_{2}O)}{J\left(D_{2}O\right)}$ , giving a constant value of 1.08 ± 0.01 from +0.1 to +1.5 V potential range (Figure 4c).

The amount of potential drop changes at the EDL δΔΦ_SCR_^L^ = −δΔΦ_EDL_^L^ leads to a change in the reaction driving force and barrier and can be analyzed to obtain further insight into the reaction kinetics. Assuming that the rate-determining step in water oxidation follows pseudo-first-order kinetics with trapped surface hole density,^19,54^ its rate constant is given by

5 $$k_{t}=k_{t,0}e^{\gamma q{\delta \Delta \Phi }_{\mathrm{SCR}}^{L}/\mathrm{kT}}$$

In eq 5, γ is an empirical coefficient representing the fraction of the increased driving force that contributes to reducing the reaction barrier, analogous to the transfer coefficient in electrode reaction kinetics,^63,64^ and *k*_*t*,0_ is the pseudo-first-order rate constant without light-induced band edge unpinning, which includes the contribution of the potential drop across the double layer in the dark. Assuming (i) recombination rate constant (*k*_rec_) is not affected by band edge unpinning at large ΔΦ_SCR_ and (ii) *k*_*t*_ + *k*_rec_ ≈ *k*_*t*_ at large ΔΦ_SCR_, the steady-state photocurrent at plateau potential range is given by eq 6:

6 $$j_{\mathrm{photo}}=qk_{t}p_{\mathrm{surf},\mathrm{ss}}=C_{\mathrm{EDL}}k_{t,0}{\delta \Delta \Phi }_{\mathrm{SCR}}^{L}e^{\gamma q{\delta \Delta \Phi }_{\mathrm{SCR}}^{L}/\mathrm{kT}}$$

Figure 4d presents the steady-state photocurrent density as a function of δΔΦ_SCR_^L^ at 1 V under varying illumination power density for H_2_O and D_2_O. Photocurrent varies nonlinearly with δΔΦ_SCR_^L^ and can be well fitted by eq 6. In H_2_O, the best fit yields *k*_*t*,0_ = 0.39 ± 0.12 s^–1^ and γ = 0.509 ± 0.033, assuming a C_EDL_ value of 50 μF/cm^2^.^47^ Fitting of the results in D_2_O by eq 6 yields *k*_*t*,0_ = 0.25 ± 0.07 s^–1^ and γ = 0.255 ± 0.020 assuming the same C_EDL_value. Both the rate constant *k*_*t*,0_ and transfer coefficient γ are smaller in D_2_O compared to those in H_2_O. Based on the rate constant *k*_*t*,0_, a larger KIE value of 1.56 ± 0.65 is reported. The dependency of reaction rate constant *k*_*t*_ on the reaction driving force can be reflected in the charge transfer coefficient γ. In H_2_O solvent, the γ value of 0.509 ± 0.033 matches the prediction from Marcus theory under modest overpotential conditions for a symmetric free energy barrier, where the rate-determining step is sensitive to the reaction driving force.^64^ A γ of 0.255 ± 0.020 is obtained in D_2_O solvent, meaning only 25% of the increased driving force is reflected in a decrease of the activation barrier. Although some errors may arise when extracting γ and *k*_*t*,0_ value as the model neglects surface recombination, it still explains the susceptibility to recombination in D_2_O solvent. The reported KIE value and KIE­(rate) are not sufficient to clearly assign whether the rate-determining step occurs through sequential proton–electron transfer (SPET) or concerted proton–electron transfer (CPET) pathway,^65,66^ where a proton-driving-force-dependent experiment may be expected to access the reaction rate constant dependency on PCET driving force. These experiments, however, are beyond the scope of our current study, as we focus on water oxidation under pH 7 unbuffered conditions. We leave this an open question for future studies. The above isotope experiments further support our assignment that δΔΦ_SCR_^L^ under rigorous stirring conditions is exclusively attributed to the accumulated photogenerated holes. Because if δΔΦ_SCR_^L^ was predominantly ascribed to local pH variation, a minimal isotopic effect on δΔΦ_SCR_^L^ would be expected, given that surface pH/pD is predominantly governed by current density and diffusion coefficient of protons/deuterons.

Further insight into the chemical nature of the rate-determining surface intermediate species can be obtained by considering the currently accepted mechanisms for the OER on TiO_2_: redox photooxidation^50,54,67,68^ and nucleophilic attack mechanisms.^69,70^ Detailed reaction pathways for these mechanisms are presented in Figure S9. Simply, in both mechanisms, oxygen evolution reaction occurs on the TiO_2_ surface via several important steps: generation of surface titanium oxo radicals, O–O bond formation, and release of surface oxygen. Cuk and co-workers have systematically studied the time scale of these key intermediate steps via time-resolved spectroscopy, where surface titanium oxo radicals form at picosecond time scale,^64,71,72^ and surface O–O bond forms at 10 μs time scale.^60^ From a kinetic perspective, further oxidation of surface peroxo species is likely to be the slowest step, i.e., the rate-determining step. In both redox photooxidation and nucleophilic attach mechanisms, surface −Ti–O–O–Ti– is proposed as the primary intermediate during the OER, with PCET as the subsequent oxidation step (Figures S9 and 5), matching the observation of our study. Thus, we postulate that surface-accumulated holes reside in the surface-bound peroxo species as the rate-determining species in pH 7 NaClO_4_, as illustrated in Figure 5. To test this assignment, we performed an EFISH control experiment in a 5% H_2_O_2_ (1.47 M) aqueous solution containing 1 M NaClO_4_ electrolyte to mimic the possible oxidation of surface −Ti–O–O–Ti– intermediate. As shown in Figure S10a, δΔΦ_SCR_^L^ acquired during the H_2_O_2_ photooxidation process is comparable with that in pure unbuffered NaClO_4_ electrolyte under a photoinduced OER process. This suggests that surface trapping of photogenerated holes also occurs during the process of H_2_O_2_ oxidation forming oxygen, with the oxidation intermediate species sharing the same microscopic origin (possibly −Ti–O–O–Ti−) as the rate-determining species during the photoinduced OER process. This surface intermediate species is regarded as the temporary surface state that gives rise to band edge unpinning on TiO_2_ in this work and also observed by others.^58,73,74^ It should be noted that our observation of surface hole trapping does not contradict the well-accepted knowledge that H_2_O_2_ acts as a fast electron donor. As long as the rate constant of rate-determining step *k*_*t*_ is large enough compared to charge recombination rate *k*_rec_, the unity hole collection efficiency can be achieved, where $\frac{k_{t}}{k_{t}+k_{\mathrm{rec}}}\to 1$ as *k*_*t*_ ≫ *k*_rec_. This control experiment supports our hypothesis of the chemical nature of OER rate-determining species in the pH 7 NaClO_4_ electrolyte. It should be noted that our measurement is only sensitive to the rate-determining step, and the OER reaction pathways can be consistent with both the redox photooxidation^50,54,67,68^ and nucleophilic attack mechanisms.^69,70^

**Figure 5:**
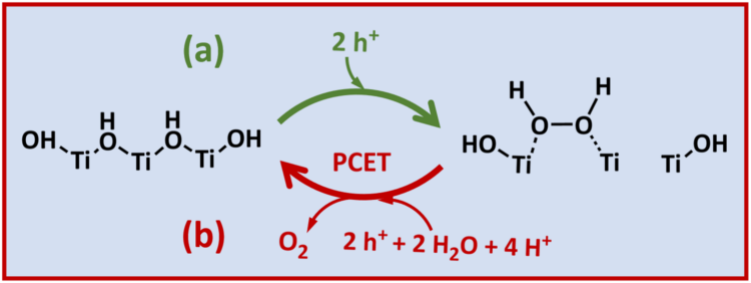
Schematic diagram of the redox photooxidation OER mechanism on TiO_2_ in pH 7 NaClO_4_, showing (a) formation of a surface-bound peroxo species and then (b) regeneration of original surface species via PCET step, accompanied with oxygen evolution.

### Effect of Electrolyte on δΔΦ_SCR_^L^ and the Reaction Mechanism

The above experiments were carried out in pH 7 NaClO_4_ solution that lacks specific adsorbing anions to compensate for surface positive charges. Previous studies have reported that surface binding electrolyte ions can alter the inner Helmholtz layer microenvironment as well as the reaction surface, not only stabilizing surface holes and reducing recombination rate^75,76^ but also altering the PCET step.^77,78^ Motivated by these observations, we also performed EFISH, photocurrent, and impedance measurements (Figure S11) in electrolytes containing specific adsorbing anions, including buffered pH 7 (1 M NaClO_4_ with 0.1 M phosphate buffer) and pH 12.7 (1 M NaClO_4_ buffered with NaOH) electrolytes. Interestingly, EFISH and Mott–Schottky responses under dark and UV illumination were nearly identical, indicating negligible surface charge accumulation and, therefore, no noticeable light-induced band edge unpinning effect. These findings imply that electrolytes with sufficiently charged surface adsorbates (e.g., HPO_4_^2–^, H_2_PO_4_^–^, and OH^–^) can compensate for the charge of the surface holes, maintaining semiconductor band bending. Beyond phosphate and hydroxide ions, charge compensation is also observed with nonbuffering, surface-adsorbing anions. For instance, replacing ClO_4_^–^ with Cl^–^, a surface-adsorbing anion on TiO_2_,^75^ results in a smaller δΔΦ_SCR_^L^ under various UV illumination power densities (Figure S10b). The decrease in δΔΦ_SCR_^L^ in the presence of HPO_4_^2–^, H_2_PO_4_^–^, OH^–^, and Cl^–^ anions should be attributed to the charge screening effect rather than an increase of rate-determining step rate constant (OER rate-determining species should still be present on the surface but not carrying charge when they are fully compensated). This is supported by the fact that the oxidation of H_2_O_2_, a well-known fast electron donor, still results in a saturated δΔΦ_SCR_^L^ of 250 mV. Given that these anions do not alter the reaction driving force (δΔΦ_SCR_^L^ = 0), it is highly unlikely that the rate-determining step reaction rate constant with the presence of surface adsorbates becomes higher than that of H_2_O_2_ oxidation. Figure 6 summarizes the electrolyte effects on δΔΦ_SCR_^L^ examined in this study.

**Figure 6:**
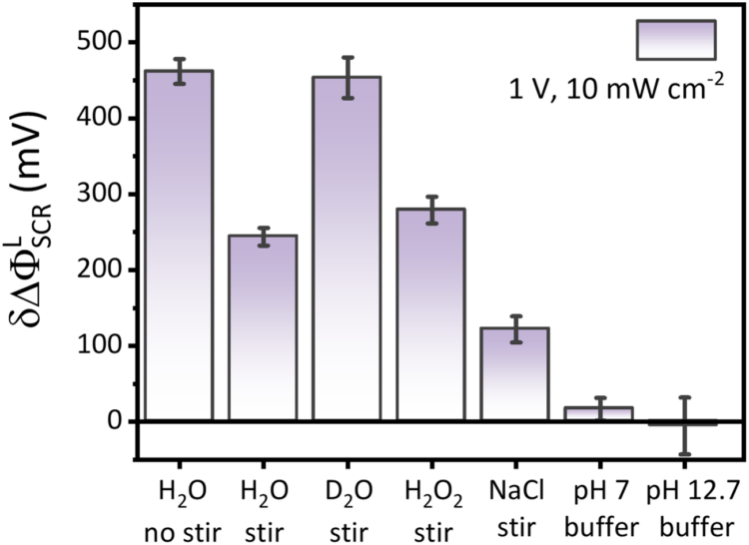
Electrolyte effects on δΔΦ_SCR_^L^ measured at 1 V vs. Ag/AgCl under 10 mW/cm^2^. Solutions pH is 7 in “H_2_O”, “D_2_O”, “H_2_O_2_”, and “NaCl” data. Except for the “NaCl” data, solutions contain a 1 M NaClO_4_ supporting electrolyte. Except for “H_2_O no stir”, “pH 7 buffer”, and “pH 12.7 buffer” data, δΔΦ_SCR_^L^ is collected under fast mass transport stirring conditions.

Modifying the solution microenvironment may also impact the reaction mechanism. While EFISH isotopic analysis (Figure 4d) is not feasible in an electrolyte that contains sufficient surface adsorbates because compensated surface-charged intermediates no longer manifest as δΔΦ_SCR_^L^. Insights can be gained by examining transient photocurrent and KIE­(rate) across different electrolytes (Figure S12). In pH 7 phosphate buffer electrolyte, band edge unpinning is mitigated, and a significantly smaller anodic transient charging current is observed at the photocurrent onset potential range (0.2–0.6 V vs. RHE) compared to an unbuffered electrolyte (Figures 3a and S12a). Additionally, a KIE­(rate) of 1 is observed. This suggests that the phosphate surface binding group compensates for surface holes and potentially alters the rate-determining reaction mechanism by acting as a hole stabilizer. Conversely, in the pH 12.7 basic solution, surface recombination remains unchanged compared to that in pH 7 unbuffered electrolytes, with the anodic charging current potential response range spanning from 0 to 0.8 V vs. RHE (Figures 3a and S12b). The observed KIE­(rate) of ∼1.05 suggests that increasing the OH^–^ concentration does not significantly alter the rate-determining reaction mechanism despite surface charge compensation reducing δΔΦ_SCR_^L^. Our observations of δΔΦ_SCR_^L^ in buffered electrolytes contrast with Hamann and co-workers’ classic impedance spectroscopy study on an α-Fe_2_O_3_ photoanode, reporting greater light-induced band edge unpinning in the pH 7 phosphate buffer electrolyte than in the pH 13.3 KOH electrolyte.^53^ However, this discrepancy is understandable given that the previous study identified different OER rate-determining species on these two photoanode materials.^20,53,65,68,69,72^

It is also interesting to consider how light-induced band edge unpinning affects the photoelectrode performance. At the potential region more negative than the photocurrent plateau potential, *i.e*., the surface recombination potential region (∼−0.5 to +0.2 V), light-induced band edge unpinning effect reduces the band bending, positively shifting both the photocurrent onset and saturation potential (Figure S13), suggesting that these surface states serve as recombination center at photocurrent onset potential region. However, at the photocurrent plateau potential range (>+0.2 V), light-induced band edge unpinning has a different effect. As shown in Figure S14, the illumination-fluence-dependent photocurrent densities were measured in different electrolytes at 1 V applied potential, and corresponding absorbed photon-to-current conversion efficiencies (APCE) were calculated via eq S10. In buffered solutions where no light-induced band edge unpinning occurs, an increase of the illumination power density from 0.5 to 20 mW/cm^2^ leads to a 9% drop of APCE: in pH 7 buffered electrolyte, ACPE decreases from 24.9 to 22.6%; in pH 12.7 buffered electrolyte, APCE decreases from 25.3 to 22.9%. This is because the reaction driving force and reaction rate constant *k*_*t*_ remain unchanged under these buffered conditions, while the surface recombination rate increases with increasing illumination fluence, leading to a decrease of APCE at higher illumination power densities. In unbuffered solutions, APCE is independent of illumination power density with values maintained at 24.4 ± 0.4% from 0.5 to 20 mW/cm^2^ illumination power density range, similar to results in Figure S8b. Under these conditions, light-induced band edge unpinning increases the surface reaction rate constant, effectively out-competing with the enhanced surface recombination at high illumination intensity. Interestingly, in an unbuffered pH 7 solution, the addition of hole scavenger H_2_O_2_ (with a much faster oxidation rate compared to H_2_O) only leads to a small increase of APCE value to 24.6 ± 0.4% at 0.5–15 mW/cm^2^ power density range. This indicates that at the photocurrent plateau potential region in unbuffered solution, bulk recombination remains as the main efficiency loss pathway, likely caused by the long penetration depth of 360 nm light and limited hole diffusion length.^79^

## Conclusions

In summary, we applied the *operando* contactless EFISH method to investigate light-induced band edge unpinning on (100) n-TiO_2_ during photoelectrochemical water oxidation. Under fast mass transport conditions in the 1 M NaClO_4_ electrolyte, band bending change in the semiconductor space charge region (δΔΦ_SCR_^L^) is observed due to photogenerated hole accumulation in surface states, agreeing well with the photoinduced band bending change determined from Mott–Schottky measurement under the same conditions. The dependence of photocurrent on light-induced band edge unpinning can be described by a model in which trapped surface holes are involved in the rate-determining step of the overall oxygen evolution reaction, with trap states acting as the oxygen evolution reaction center. Kinetic isotope experiments establish that the rate-determining reaction step of the trapped holes involves a PCET process, agreeing well with the previously proposed surface peroxo species. We further demonstrated that δΔΦ_SCR_^L^ can be mitigated by modifying the microenvironment of the electric double-layer, where surface adsorbates compensate for hole accumulation and potentially alter the reaction mechanism. While δΔΦ_SCR_^L^ affects the photocurrent at the onset region, requiring greater band bending for effective charge separation under the same illumination power density, it maintains the plateaued APCE at higher illumination power density by increasing the water oxidation rate constant *k_t_*, which is important in actual device applications. This study demonstrates that EFISH is a versatile *operando* technique for investigating the light-induced change of the junction electrostatic potential profile during the photoelectrochemical process and for providing insights into the chemical nature of rate-determining species.

## Supplementary Material



## Notes

Full experimental methods, supplementary Notes S1–S4, supplementary Figures S1–S14, and Table S1 (PDF)
