# Supplementary material for: Operando Contactless EFISH Study of the Rate-Determining Step of Light-Driven Water Oxidation on TiO2 Photoanodes
Source: J Am Chem Soc. 2025 May 21;147(22):18712–22. doi: 10.1021/jacs.5c01836 (PMC12147135; doi:10.1021/jacs.5c01836)
Supplement: Supplementary file 1 [file ja5c01836_si_001.pdf]

Supporting Information for

***Operando* Contactless EFISH Study of the Rate-determining Step of Light-driven Water Oxidation on TiO_2 Photoanodes**

Fengyi Zhao[a], Zihao Xu[a], Sa Suo[a], Yixuan Xu[a], Craig L. Hill[a], Djamaladdin G. Musaev[a,b], Tianquan Lian[a*]

[a]Department of Chemistry, Emory University, Atlanta, GA, 30322, USA

[b]Cherry L. Emerson Centre for Scientific Computation, Emory University, 1515 Dickey Drive, Atlanta, GA, 30322, USA

Table of Contents

1. Experimental Section....................2

1.1. Materials....................2

1.2. Experimental methods....................2

2. Supplementary Notes....................3

Note S1. Reference potential conversion....................3

Note S2. Mott-Schottky analysis and surface charge capacitance measurements....................4

Note S3. Analysis of local pH change....................5

Note S4. Absorbed photon-to-current efficiency (APCE) calculation....................6

3. Supplementary Figures S2-S14 and Table S1....................6

1. Experimental Section

1.1. Materials

A Nb-doped rutile TiO_2 (100) (wt 0.5%) with a dopant concentration of ~10^{20} cm^{-3} (1 cm by 1 cm, 0.5 mm thickness, MTI Corporation) was used in our study. We expose the (100) surface following a previously reported procedure, where the electrode is photoelectrochemically etched to generate a clean surface in 0.1 M H_2SO_4 a 2 V vs. Ag/AgCl applied bias under a 150 W Xe lamp for 1 h before the EFISH and electrochemical measurement.[1, 2]

Aqueous solutions are used in the experiments in this work, where 18 MΩ cm Milli-Q water and deuterium oxide (Aldrich Chemistry), respectively, were used as solvents. H_2O is the default solvent unless otherwise mentioned. 1 M $NaClO_4$ (Acros Organics, 99+% for analysis) is used as supporting electrolyte. 0.1 M pH 7 phosphate buffer is obtained by adding 1.549 g sodium phosphate dibasic heptahydrate (Macron Chemicals) and 0.583 g sodium phosphate monobasic monohydrate (Macron Chemicals) to 100 mL 1 M $NaClO_4$ solution. pH 12.7 solution is obtained by titrating 1 M $NaClO_4$ with 1 M NaOH (Macron Fine chemicals) solution until the pH reaches 12.7. Sodium chloride (Macron Chemicals), and hydrogen peroxide solution (Sigma-Aldrich, 30 wt. % in H_2O) are used as solutes for different control experiments.

1.2. Experimental methods

***Operando* EFISH measurement.** The Second Harmonic Generation measurements were measured by a Bialkali Amplified Photomultiplier Tube (PMT) detector (Thorlabs, PMM01) with tube voltage powered by a 1.3 V DC power supply (GW INSTEK, GPD-230S). A 3mW, 1 kHz 800 nm pulse is used as the SHG fundamental light. The fundamental is derived from a regenerative amplified Ti: sapphire laser system (Coherent Astrella, 800 nm, 5 mJ/pulse, 35 fs pulse width, and 1 kHz repetition rate). The fundamental pulse energy is tuned by a variable neutral density filter (Thorlabs, NDC-50C-4M-A) to 3 μJ and further focused down to a beam size with a waist of 100 μm at the sample surface with an angle of incident of 45 degrees using a f = 100 mm lens (Thorlabs, LBF254-100-A). The SHG signal is collected and collimated using a lens with f = 50 mm (Thorlabs, LBF254-050-A) and passes two 400 nm bandpass filters (Thorlabs, FBH400-10) before reaching the PMT detector. The polarization of fundamental

pulse is controlled by a half-wave plate (Thorlabs, AQWP05M-600) before focus and polarization of SHG pulse is selected by a linear polarizer (Thorlabs, LPVISE100-A) after collimation lens and before bandpass filter. The signal is further processed by a boxcar averager (Stanford Research SR200 Series) for noise filtering. The gated signal is averaged 1000 times by the boxcar average and sent to a data acquisition board, read out by homebuilt LabVIEW software. The collected voltage signal is transformed into SHG counts using the transimpedance gain and responsivity parameter provided by the manufacturer.

Illumination light source. Monochromic 360 nm continuous-wave (CW) light is provided by a 365 nm LED (Thorlabs, M365 L3) with a 360 nm bandpass filter (Thorlabs, FB360-10, FWHM=10 $\pm$ 2 nm). The LED is controlled by a DC driver (Thorlabs, DC2200). The 360 nm CW light illuminates the sample at a normal angle of incident with a beam area of 0.055 cm^2, overlapping with the 800 nm fundamental probe.

(Photo)electrochemical measurement. *Operando* EFISH and electrochemical measurements are conducted in a three-electrode system as shown in Figure 2a, using a graphite electrode as a counter electrode and Ag/AgCl (1 M KCl) as a reference electrode. Electrochemical measurements are performed using a CHI 660e electrochemical workstation (CH Instruments). All cyclic voltammetry and linear sweep voltammetry presented in this study are measured using a scan rate of 3.53 mV/s unless stated otherwise. A stirring rate of 1500 rpm is applied to the solution by a Teflon stir bar. Impedance measurements are measured under the negative scan direction with a 550 Hz AC frequency with a 0.005 V amplitude. AC impedance measurement is done by scanning frequency from 100 kHz to 1Hz under a fixed potential with a 0.005 V of perturbation AC voltage.

2. Supplementary Notes

Note S1. Reference potential conversion

Reference potentials can be converted to RHE scale from Ag/AgCl (filled with 1M KCl) scale following Eq. (S1):

$$E_{RHE} = 0.235 + E_{Ag/AgCl} + 0.059 * pH \quad (S1)$$

Note S2. Mott-Schottky analysis and surface charge capacitance measurements

Conventional electrochemical impedance spectroscopy is adopted to confirm the light-induced band edge unpinning phenomenon observed in EFISH measurement. The Mott-Schottky measurement on TiO_2 was conducted under the same conditions as EFISH measurement (1 M $NaClO_4$, rigorously stirred solution) with and without CW illumination to extract the flatband potential (U_{fb}^{D}) and band unbending $\delta\Delta\Phi_{SCR}^{L}$ using Eq. (S2) as shown in Figure S3:[3-8]

$$\frac{A^2}{C_s^2} = \frac{2}{q\varepsilon\varepsilon_0 N_d}[(U_{app} - U_{fb}^{D}) - \delta\Delta\Phi_{SCR}^{L} - \frac{kT}{q}] \tag{S2}$$

where N_d is the doping density of the semiconductor, C_s and A are the interfacial capacitance and electrode area, respectively, q is the elementary charge, ε_0 and ε are the vacuum dielectric constant and the relative dielectric constant of the semiconductor, respectively, k is Boltzmann's constant, and T is the temperature.

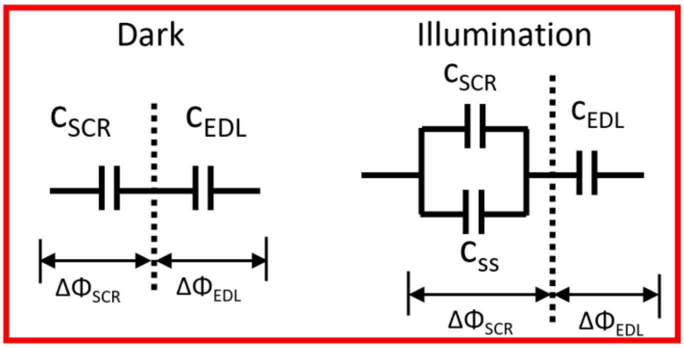

Figure S1. Simplified equivalent circuit of semiconductor/electrolyte with and without illumination, only showing the capacitance component.

As shown in Figure S1, under the dark conditions, capacitance of semiconductor space charge region (C_{SCR}) and electric double layer (C_{EDL}) are in series, and the total capacitance can be written as Eq. (S3):

$$C_{tot} = \frac{C_{SCR}C_{EDL}}{C_{SCR} + C_{EDL}} = \frac{C_{SCR}}{1 + \frac{C_{SCR}}{C_{EDL}}} \tag{S3}$$

As $C_{EDL} \gg C_{SCR}$ can be assumed under the high electrolyte concentration used in this study, semiconductor capacitance dominates the overall capacitance response, i.e., $C_{tot} \approx C_{SCR}$.

Under illumination, surface charge accumulation leads to photogenerated parallel surface charge capacitance C_{ss}. The overall capacitance response of semiconductor/electrolyte junction can be approximate as $C \sim (C_{SCR} + C_{ss})$.[9-14] By subtracting the capacitance under illumination from that in the dark, the capacitance response of photo-induced surface charge (C_{ss}) can be calculated.

The C_{ss}-potential response can be further fitted by Gaussian distribution of surface states as shown in Eq. (S4):[6, 15, 16]

$$C_{ss} = \frac{qN_{ss}}{\sqrt{2\pi}\sigma} \exp\left[-\frac{(U - U_{ss})^2}{2\sigma^2}\right] \quad (S4)$$

where N_{ss} is the surface charge density, σ and U_{ss} are the standard deviation and peak potential of the surface state response, respectively.

Note S3. Analysis of local pH change

In an unbuffered pH 7 solution, surface local pH variation caused by photoelectrochemical OER ($2H_2O - 4e^- \rightarrow O_2 + 4H^+$) can be calculated using the following model, where Eqs. S6-S8 are originally derived by Zhao and co-workers:[17]

The relationship between OER current density and H^+ and OH^- concentration gradient can be described by:

$$D_{OH^-} \cdot \frac{d[OH^-]}{dx} - D_{H^+} \cdot \frac{d[H^+]}{dx} = \frac{J_{ph}}{F} \quad (S5)$$

where $[H^+]$, $[OH^-]$ denote the concentration of H^+ and OH^- separately, in until of mol· m^{-3}; D_{OH^-} and D_{H^+} are diffusion coefficients of OH^- (5.3×10^{-9} $m^2 \cdot s^{-1}$) and H^+ (9.3×10^{-9} $m^2 \cdot s^{-1}$), separately; x is the distant from the electrode surface, F is the Faraday constant (F = 96485 $A \cdot s \cdot mol^{-1}$).

Replacing the $[OH^-]$ using equilibrium constant (K_w) in water, $[H^+]$ can be solved by assuming an effective diffusion layer thickness of δ:

$$[H^+] = -\frac{A}{2} + \sqrt{\left(\frac{A}{2}\right)^2 + B} \quad (S6)$$

where

$$A = \frac{D_{OH^-} \cdot K_w}{D_{H^+} \cdot [H^+]_{bulk}} - [H^+]_{bulk} - \frac{J_{ph} \cdot \delta}{F \cdot D_{H^+}} \tag{S7}$$

$$B = \frac{D_{OH^-} \cdot K_w}{D_{H^+}} \tag{S8}$$

$\delta\Delta\Phi_{\mathrm{SCR}}^{\mathrm{L}}$ induced by surface pH change can be calculated by Nernst equation:

$$\delta\Delta\Phi_{\mathrm{SCR}}^{\mathrm{L}}(\mathrm{mV}) = \left(pH(bulk) - pH(surface)\right) \times 59 \tag{S9}$$

Note S4. Absorbed photon-to-current efficiency (APCE) calculation

Chop-light chronoamperometric measurement is conducted to more accurately determine the steady state photocurrent densities and APCE, where APCE can be calculated using the following Eq. (S10):[18]

$$\mathrm{APCE} = \frac{J_{inf} \times 1239.8}{P_{mono} \times \lambda \times (1 - T - R)} \times 100\% \tag{S10}$$

where J_{inf} (mA cm^{-2}) is the steady-state photocurrent density; P_{mono} (mW cm^{-2}) is the power density of monochromatic incident light; T and R are the ratio of transmitted and reflected light, respectively. In our study, the reflection loss is calculated to be 13.8% using the Fresnel equation and T is 0; λ (nm) is the wavelength of monochromatic light; and 1239.8 (V nm) is a coefficient from the multiplication of speed of light, reciprocal of unit charge and Planck's constant.

3. Supplementary Figures S2-S14 and Table S1

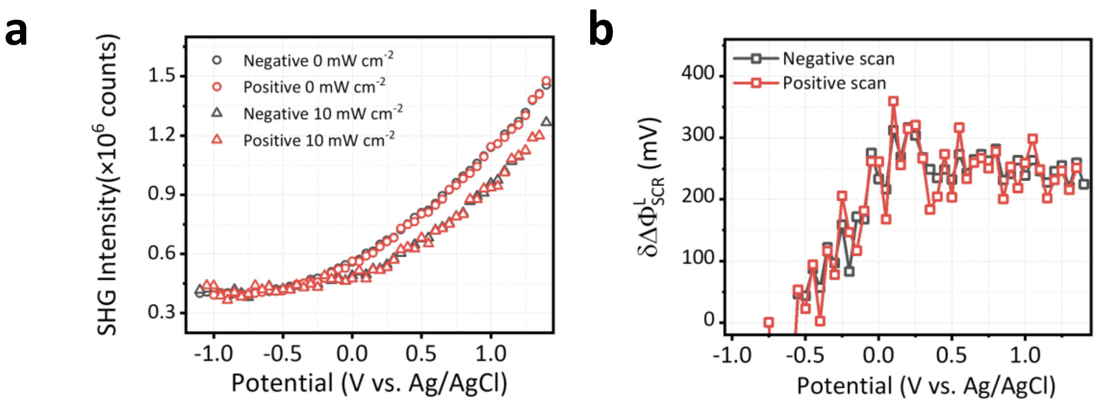

Figure S2. (a) TiO_2 EFISH intensity as a function of applied potential under 0 (open circle) and 10 (open triangle) mW/cm^2 360 nm illumination at negative (grey) and positive (red) potential scan direction. (b) Calculated $\delta\Delta\Phi^{L}_{SCR}$ as a function of applied potential under 10 mW/cm^2 illumination under negative (grey square) and positive (red square) potential scan direction.

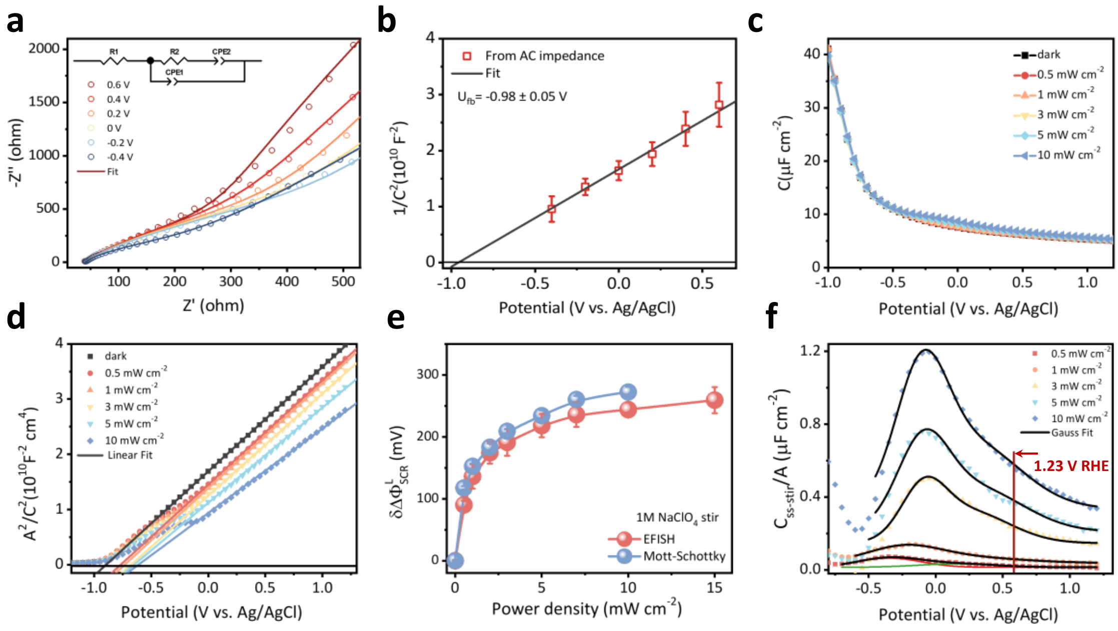

Figure S3. Impedance measurement of TiO_2 photoanode. (a) Nyquist plot of AC impedance under dark conditions, the inset shows the equivalent circuit Nyquist plot is fitted to (from 5 Hz to 50 kHz). (b) Mott-Schottky plot using SCR capacitance extracted from the Nyquist plot fit in (a). (c) Impedance-potential measurement of TiO_2-electrolyte junction under varying illumination power densities with 550 Hz AC voltage perturbation. (d) Mott-Schottky plot extracted from junction impedance-potential measurement in (c). (e) Comparison of $\delta\Delta\Phi^{L}_{SCR}$ measured by Mott-Schottky (blue trace) and by EFISH (red trace, extracted at 1 V) as a function of illumination power density. (f) Surface state capacitance as a function of applied potential under different illumination power densities, where formal water oxidation potential is indicated. The surface state capacitance in (f) is calculated by subtracting capacitance measured under illumination conditions from that in the dark in (c) (details in Note S2), the surface state capacitance is fitted using Gaussian distribution from (U^{D}_{fb} + 0.2) V to 1.2 V vs. Ag/AgCl. A fitting example at 0.5 mW cm^{-2} is plotted. All fitting details are summarized in Table S1. All the above experiments were done in 1 M $NaClO_4$ electrolyte under stirring conditions.

As shown in Figure S3, AC impedance and impedance-potential measurements were used to characterize the electrochemical properties of TiO_2-electrolyte junction. In AC

impedance measurements, a frequency sweep from 100 kHz to 1 Hz was performed under dark conditions to generate Nyquist plot at a specific applied potential. A previously reported equivalent circuit (Figure S3a inset) was used to fit the Nyquist plots.[19] Here, constant phase element (CPE) 1 represents semiconductor SCR capacitance, CPE 2 represents surface state capacitance, R1 and R2 denotes circuit resistance and SCR resistance, respectively. Since no charge transfer occurs under dark conditions, the resistor element associated with the surface-state Randles circuit was omitted. Values of $1/C^2$ (using values of CPE 1 element) were extracted from Nyquist plot fitting at varying applied potentials. Using Eq. (S2), the flatband potential under dark conditions was calculated at -0.98 $\pm$ 0.05 V. Mott-Schottky plot can also be extracted from a conventional impedance-potential measurement at a single frequency (550 Hz) as shown in Figure S3d, for which a U_{fb} of -0.926 $\pm$ 0.001 V was determined under dark conditions. This value agrees with the U_{fb}^{D} values obtained from AC impedance measurement (Figure S3b) and EFISH measurement (Figure 2c) within the error range. This consistency confirms that impedance measurements at 550 Hz reflect mainly the SCR impedance in depletion potential range. For conveniency, impedance data acquired at 550 Hz were used to analyze light-induced band edge unpinning effect (Figures 3d, 4d, S11a-b) in the following studies.

As shown in Figure S3d, under illumination conditions, TiO_2 Mott-Schottky curve exhibits a shift towards positive potential, while maintaining similar slopes of the linear part (at photocurrent saturation potential region shown in Figure 2b). In the conventional interpretation, this means that the flatband potential changes under illumination. However, as shown in Figures 2c and d, $\delta\Delta\Phi_{SCR}^{L}$ is a bias-dependent variable. In this scenario, we only fit the linear part of the Mott-Schottky plot under light illumination. By comparing the difference between “hypothetical flatband potential” and U_{fb}^{D} under dark condition, we extract the saturated $\delta\Delta\Phi_{SCR}^{L}$ value under a certain illumination power density.

The shift of “hypothetical flatband potential” is attributed to the trapping of minority carriers in surface states or surface modification upon adsorption of solution species or electrode corrosion in previous studies.[13, 20-25] In this study, we believe the surface modification or electrode corrosion are less likely, as TiO_2 is rather robust against photo-corrosion and the $NaClO_4$ electrolyte does not exhibit an adsorption effect on TiO_2 surface. The saturated $\delta\Delta\Phi_{SCR}^{L}$ extracted from both EFISH and Mott-Schottky method changes with UV power density in a very similar way as shown in Figure S3e. This confirms that EFISH measures the

same light-induced Fermi level pinning effect as conventional Mott-Schottky measurement does. In contrast to Mott-Schottky analysis, however, that only extracts the saturated $\delta\Delta\Phi_{SCR}^{L}$, the EFISH method has the capability of probing the bias-dependent and time-dependent $\delta\Delta\Phi_{SCR}^{L}$ behavior, making it a more *operando* methodology.

The surface state capacitance (C_{ss}) can be extracted by subtracting the capacitance under illumination (C_{light}) from that in the dark (C_{dark}) in Figure S3c and plotted as a function of applied potential as shown in Figure S3f. The surface state capacitance can be further fitted by Eq. (S4) with fitting results summarized in Table S1, where two trap states potential U_{ss1} and U_{ss2} can be found.

Interestingly, U_{ss} of both trap states exhibit a similar positive shift as a function of illumination power density similar to the trend in $\delta\Delta\Phi_{SCR}^{L}$ shown in Figure S3e. This agrees with the physical picture that all surface energy levels shift with the band edge position at the surface in response to electrostatic potential redistribution across the semiconductor/electrolyte junction. Moreover, the potentials of these trap states agree well with the photocurrent onset region, i.e., from -0.5 to +0.2 V, where increased band bending suppresses the surface recombination. This suggests that hole-trapping surface states are also responsible for surface recombination when there is insufficient band bending on electrode. What needs to be mentioned is that although a CPE element representing surface charge capacitance is included to fit the Nyquist plot (Figure S3a) measured under dark conditions, the physical meaning of this surface state capacitance is different from the light-induced surface states analyzed in Note S2 and Figure S3f, where the former refers to the trap states that interact with the electron Fermi level when no OER occurs, and the later refers to the temporary states that originates from the accumulated OER intermediates under light-induced OER conditions.

Although the measured trap states capacitance potential (ca. 0.5 V for U_{ss1} and 0.9 V vs. RHE for U_{ss2} at 10 mW/cm^2 for example) does not meet the formal water oxidation potential at 1.23 V vs. RHE, this does not mean the reaction does not occur through the surface states. This is because the observed trap states position obtained from impedance measurement is determined not only by the trap states hole/electron capture/emission rate with semiconductor conduction band/valance band, but also by hole/electron transfer rate from surface states to solution.[26] Surface state potential revealed by the quasi-Fermi level of electrons, i.e., applied potential, do not reflect the energy of quasi-Fermi level of holes, which is the free energy that dictates the reactivity of the reaction[27]. Electroluminescence studies can provide an alternative

way to reveal the surface state energy that relates to quasi-Fermi level of holes, where an energy level of 1.47 eV below the conduction band edge is measured on TiO_2 under illumination condition.[16, 28, 29] More importantly, under the steady-state condition, reaction center energetic needs to fulfill the rate-determining reaction energy level rather than the formal OER reaction energy level. The energy level that is 1.47 eV below the conduction band edge should easily satisfy the oxidation of surface-bound proxo species, a surface-bound rate-determining species proposed from the analysis of Figures 4 and 5.

Table S1. Gauss fitting result of surface state capacitance C_{ss} response from Figure S3f.

Power density (mW/cm^2)	0.5	1	3	5	10
**U_{ss1} (V vs. AgAgCl)**	-0.353 ± 0.020	-0.235 ± 0.019	-0.092 ± 0.008	-0.096 ± 0.010	-0.107 ± 0.005
**σ_1 (V)**	0.211 ± 0.009	0.247 ± 0.029	0.163 ± 0.026	0.209 ± 0.050	0.189 ± 0.022
**N_{ss1} (10^{11}cm^{-2})**	1.60 ± 0.23	2.90 ± 1.15	6.65 ± 3.70	13.60 ± 12.60	19.00 ± 7.80
**U_{ss2} (V vs. AgAgCl)**	0.099 ± 0.008	0.097 ± 0.340	0.239 ± 0.158	0.293 ± 0.423	0.258 ± 0.164
**σ_1 (V)**	0.275 ± 0.055	0.509 ± 0.076	0.303 ± 0.090	0.396 ± 0.264	0.365 ± 0.098
**N_{ss2} (10^{11}cm^{-2})**	0.83 ± 0.30	3.55 ± 0.94	8.26 ± 0.46	14.10 ± 18.30	20.7 ± 10.6

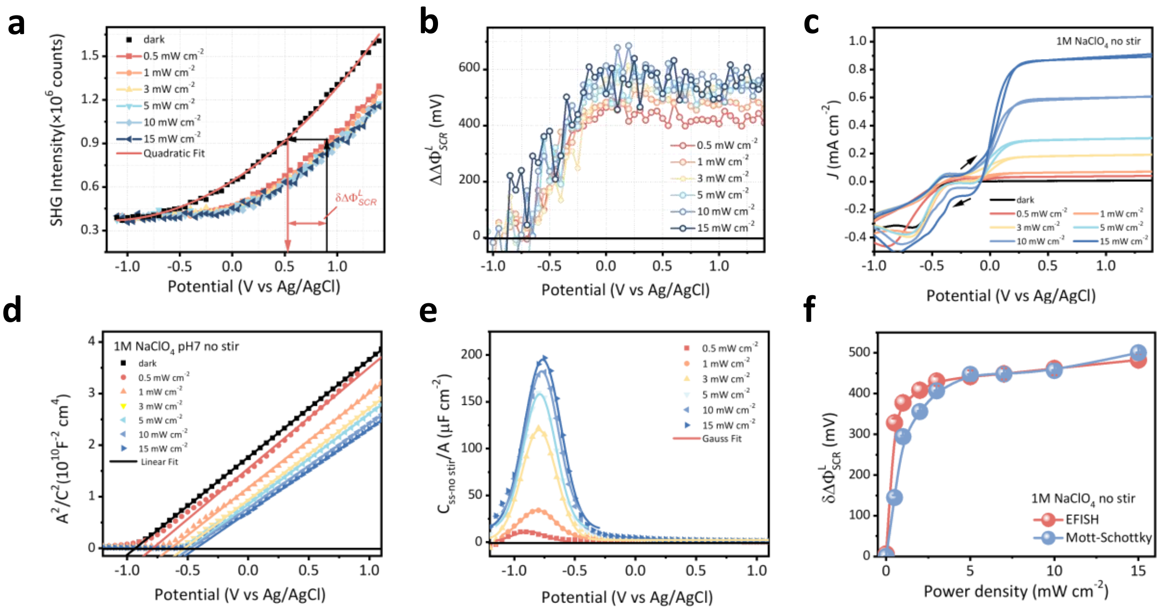

Figure. S4. EFISH and impedance measurement of TiO_2/electrolyte junction with no solution stirring. (a) EFISH intensity, (b) $\delta\Delta\Phi_{SCR}^{L}$, (c) current densities, (d) Mott-Schottky plot, and (e) surface charge capacitance $C_{ss\text{-}no\ sitr}$ as a function of applied potential under different illumination power densities (extracted the same way as C_{ss} in Figure S3f). (f) $\delta\Delta\Phi_{SCR}^{L}$ measured by Mott-Schottky (blue) and by EFISH (red, extracted at 1 V) as a function of illumination power density. Both EFISH and impedance measurement are conducted under negative scan direction, and 1 M $NaClO_4$ is used as the electrolyte.

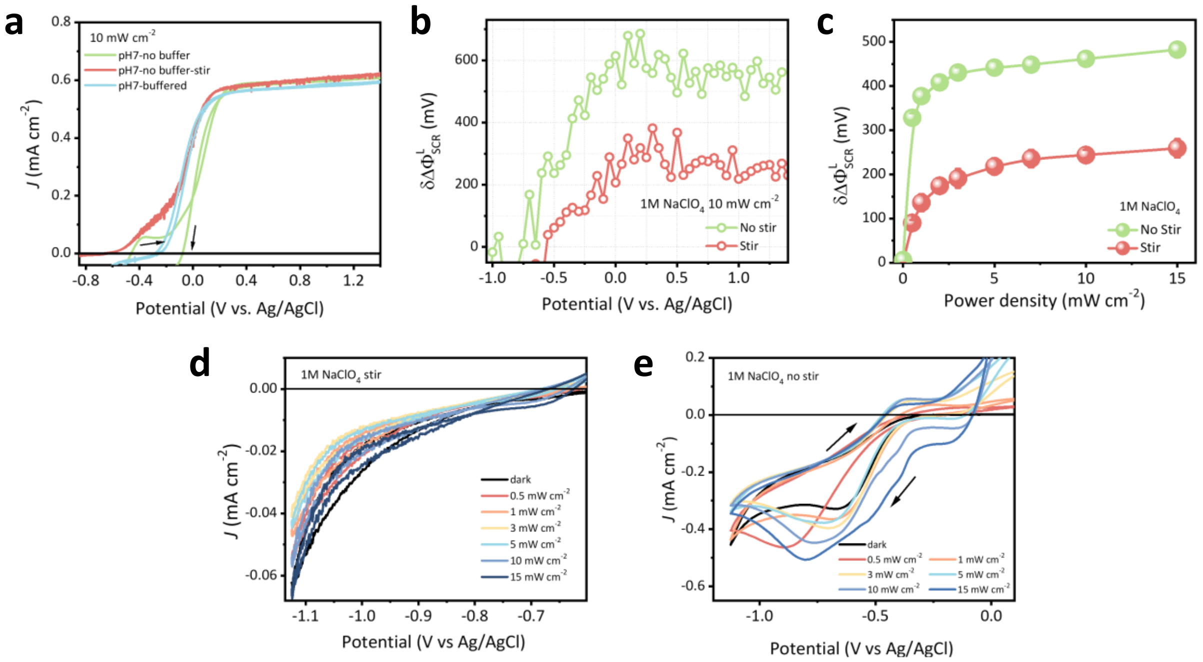

Figure. S5. Stirring control experiment. (a) CV current on TiO_2 measured in pH 7 1 M $NaClO_4$ solution with (red) and without (green) stirring conditions, and in 0.1 M pH 7 sodium phosphate buffer with stirring (blue) under 10 mW/cm^2 illumination. (b) $\delta\Delta\Phi_{SCR}^{L}$ plot as a function of applied potential with (red) and without (green) stirring under 10 mW/cm^2 illumination. (c) $\delta\Delta\Phi_{SCR}^{L}$ (collected at 1V) as a function of illumination intensity with (red) and without (green) stirring. Cathodic CV current on TiO_2 under varying illumination intensity in 1 M $NaClO_4$ solution with (d) stirring and (e) no stirring applied.

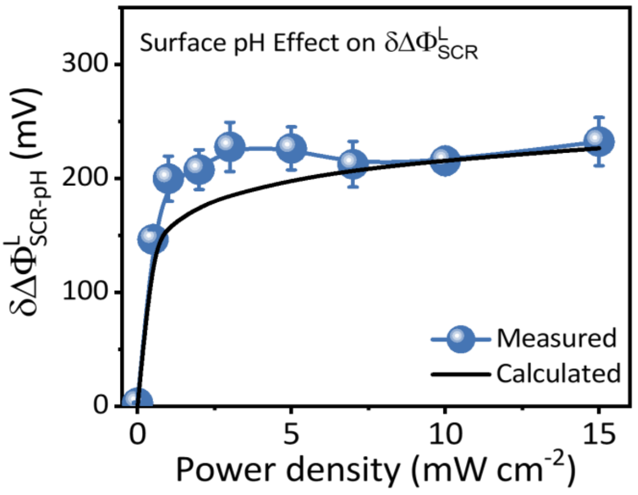

Figure. S6. $\delta\Delta\Phi^{\mathrm{L}}_{\mathrm{SCR-pH}}$ induced by surface pH effect, where measured $\delta\Delta\Phi^{\mathrm{L}}_{\mathrm{SCR-pH}}$ is calculated by subtracting $\delta\Delta\Phi^{\mathrm{L}}_{\mathrm{SCR}}$ under stirring condition (only hole trapping) from $\delta\Delta\Phi^{\mathrm{L}}_{\mathrm{SCR}}$ under no stirring condition (pH and hole trapping effect), which are extracted from Figures S4f and S3e, respectively. $\delta\Delta\Phi^{\mathrm{L}}_{\mathrm{SCR-pH}}$ can also be estimated based on Eqs. (S5-9) using the photocurrent density. In the calculation, an effective diffuse layer $\delta = 70$ μm is assumed, and photocurrent densities (J_{ph}) parameter under 1 V applied potential and various illumination power densities are extracted from Figure S4c under no stirring conditions.

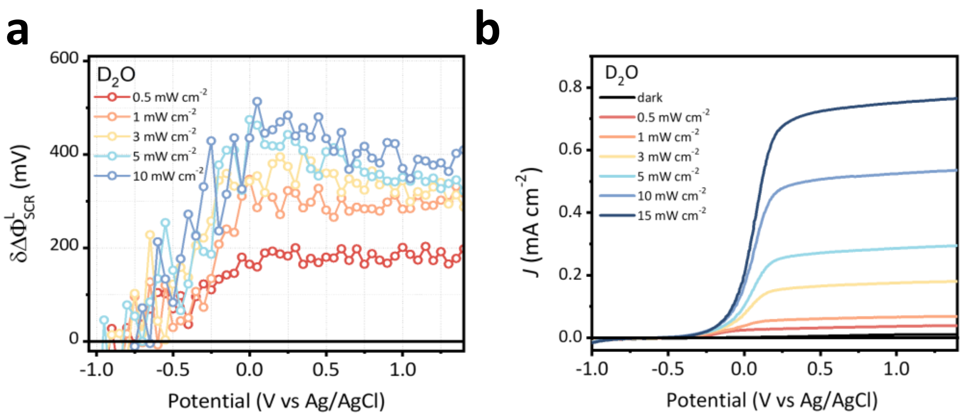

Figure. S7. KIE control experiment. (a) $\delta\Delta\Phi^{\mathrm{L}}_{\mathrm{SCR}}$ and (b) LSV current densities measured in D_2O solvent containing 1 M $NaClO_4$ supporting electrolyte under varying illumination power densities. Both EFISH and LSV experiments are performed under negative scan direction under solution stirring conditions.

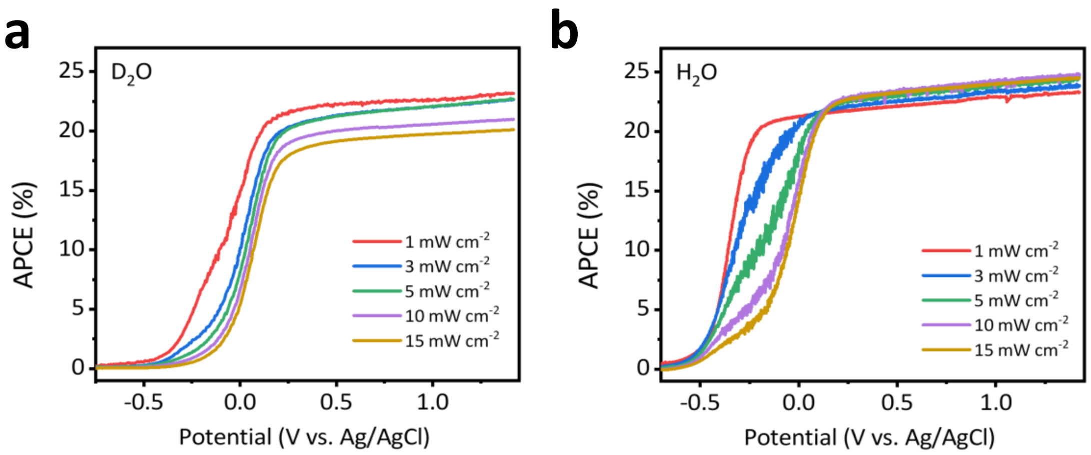

Figure. S8. APCE calculated under varying illumination intensities in (a) D_2O and (b) H_2O containing 1 M $NaClO_4$ as supporting electrolyte based on negative scan photocurrent shown in Figures. S7b and 2b, respectively. APCE is calculated using Eq. (S10).

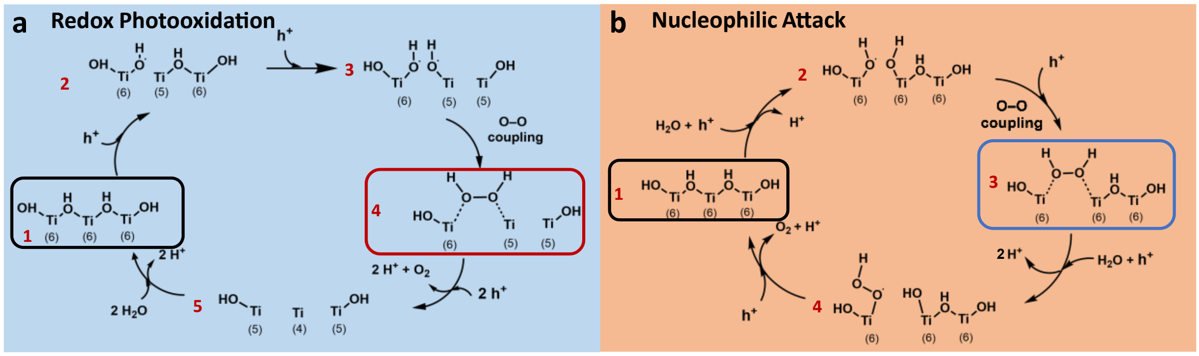

Figure. S9. Summary of (a) redox photooxidation (RP) and (b) nucleophilic attack water oxidation mechanisms on TiO_2 surface.[30] In each mechanism, the catalytic cycle starts with species **1**, with **4** in RP mechanism and **3** in NA mechanism being the proposed rate-determining species. Coordination number is labeled under each Ti atom. (Reproduced with permission, Copyright 2010 Elsevier)

According to the redox photooxidation (RP) mechanism proposed by Salvador et al. and others,[16, 30-34] the photogenerated holes are trapped at the two-fold coordinated bridging oxygen atom, leading to the breaking of Ti-O bond and the generation of one-fold coordinated hydroxyl radical Ti-OH·; two adjacent Ti-OH· form a bridging hydrogen peroxide; further oxidation of surface hydrogen peroxide by two holes leads to the evolution of an oxygen

molecule and the release of two protons; and finally, adsorption of water molecules on hydroxyl vacancies, regenerates the bridging hydroxyl ions. According to nucleophilic attack (NA) mechanism of Nakato et al and others,[35-38] water molecules act as a Lewis base, attacking the initial surface-trapped hole and forming hydroxyl radicals; trapping of another hole on the surface forms a surface oxygen peroxide bound to two adjacent Ti-terminated sites; a nucleophilic attack of surface-bound hydrogen peroxide by water releases oxygen and protons and regenerates the Ti-O bridging sites. Interestingly, in both mechanisms, the surface-bound hydrogen peroxide species is proposed, confirmed by observation of O-O stretching mode via *in situ* multiple internal reflection infrared (MIRIR) spectroscopy.[35]

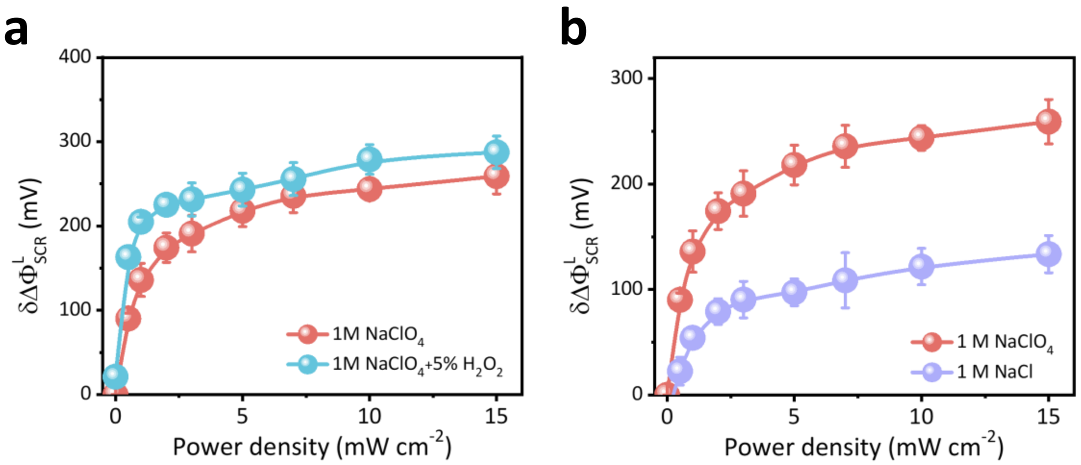

Figure. S10. Power dependent $\delta\Delta\Phi^{L}_{SCR}$ comparison collected in between (a) 1 M $NaClO_4$ and 1 M $NaClO_4$ containing 5% H_2O_2 (blue) electrolyte, and (b) 1 M $NaClO_4$ (red) and 1 M NaCl (purple). $\delta\Delta\Phi^{L}_{SCR}$ is measured at 1 V vs. Ag/AgCl applied potential under stirring conditions.

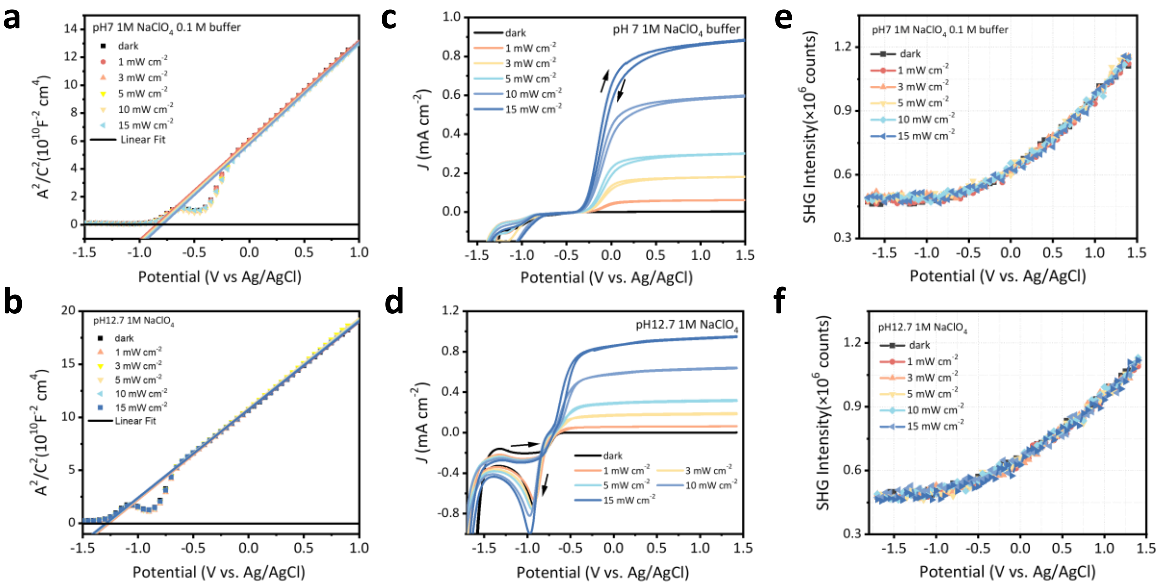

Figure. S11. Mott-Schottky plot in (a) pH 7 with 0.1 M phosphate buffer and (b) pH 12.7 buffered solution. *J*-V curve in (c) pH 7 with 0.1 M phosphate buffer, and (d) pH 12.7 buffer solution. Bias-dependence EFISH measured in (e) pH 7 with 0.1 M phosphate buffer, and (f) pH 12.7 buffered solution. All buffer solutions contain 1 M $NaClO_4$ supporting electrolyte. Data were acquired under varying UV illumination power densities and without solution stirring as surface pH is stabilized by buffer solution.

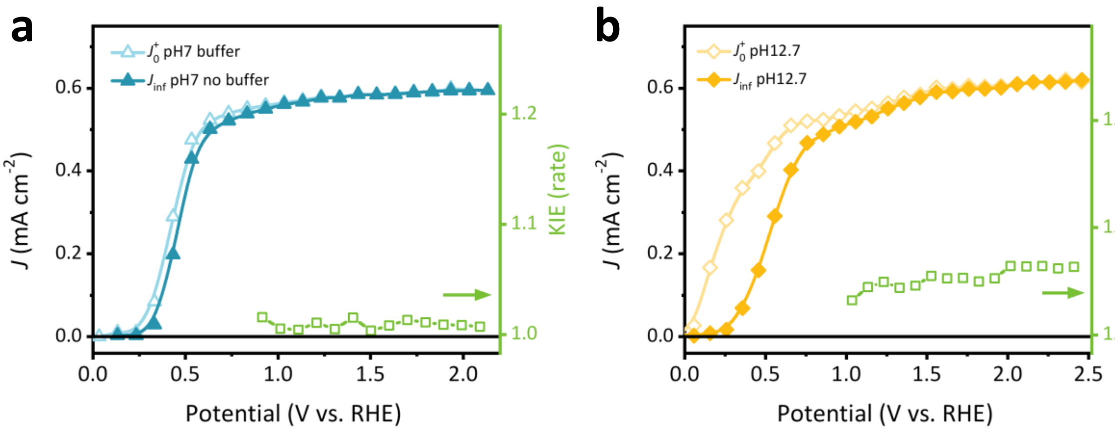

Figure. S12. Transient photocurrent J_0^+ and J_{inf}, and KIE (rate) on TiO_2 under 10 mW/cm^{-2} in (a) pH 7 buffer and (b) pH 12.7 buffer solution. The experiments are performed with chop-light chronoamperometric measurement under stirring conditions. KIE (rate) is calculated based on J_{inf} value.

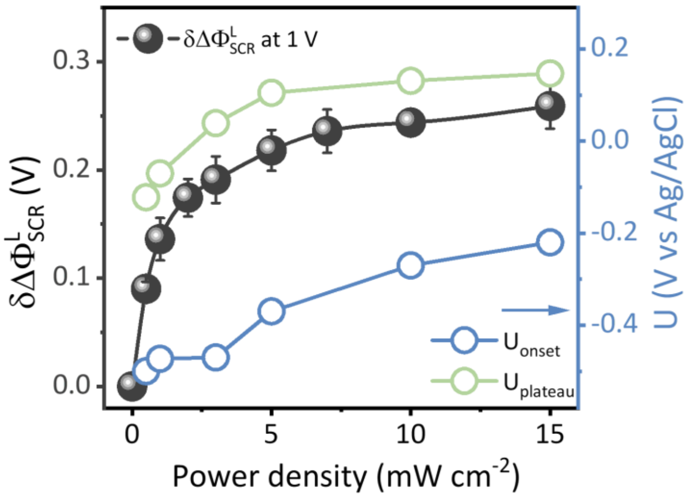

Figure. S13. Effect of UV illumination power density on $\delta\Delta\Phi^L_{SCR}$, photocurrent onset potential (U_{onset}), and photocurrent plateau ($U_{plateau}$) potential in 1 M $NaClO_4$ electrolyte (unbuffered) with stirring applied. U_{onset} and $U_{plateau}$ are extracted from Fig. 2b, where U_{onset} is defined by the potential cross point between the tangent of photocurrent at current inflection region and the extrapolation of the exchange current baseline,[39] $U_{plateau}$ is defined as the potential where photocurrent reaches 90 % of its value obtained at 1 V.

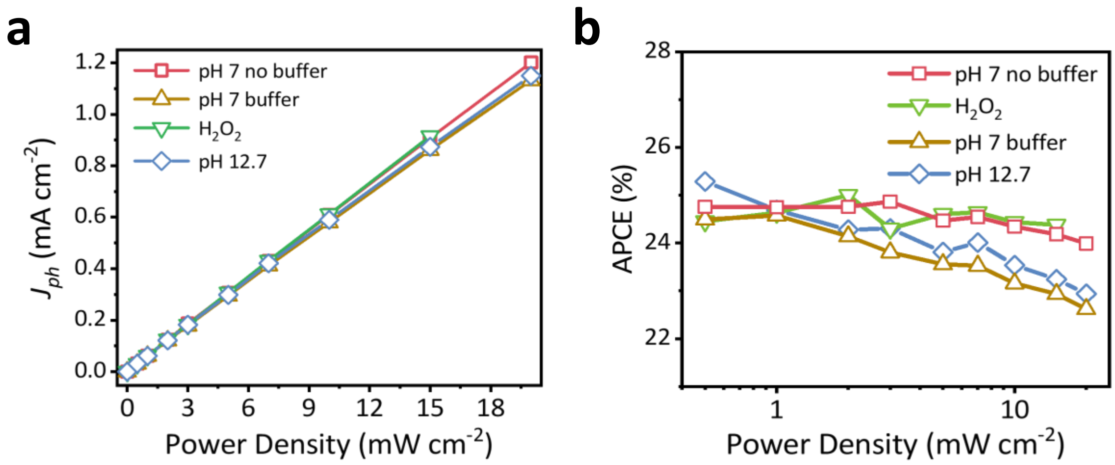

Figure. S14. (a) Photocurrent at 1 V in different electrolytes and (b) corresponding APCE calculated following Eq. (S10). pH 7 buffer solution contains 0.1 M phosphate buffer, pH 12.7 solution is buffered with NaOH, H_2O_2 solution is 5 % H_2O_2 solution. All solutions contain 1 M $NaClO_4$ as supporting electrolyte. Photocurrents were measured under stirring conditions.

References

(1) Lu, Y.; Jaeckel, B.; Parkinson, B. A. Preparation and Characterization of Terraced Surfaces of Low-Index Faces of Anatase, Rutile, and Brookite. *Langmuir* **2006**, *22* (10), 4472-4475.
(2) Tsujiko, A.; Kisumi, T.; Magari, Y.; Murakoshi, K.; Nakato, Y. Selective Formation of Nanoholes with (100)-Face Walls by Photoetching of n-TiO_2(Rutile) Electrodes, Accompanied by Increases in Water-Oxidation Photocurrent. *The Journal of Physical Chemistry B* **2000**, *104* (20), 4873-4879.
(3) De Gryse, R. On the Interpretation of Mott-Schottky Plots Determined at Semiconductor/Electrolyte Systems. *Journal of The Electrochemical Society* **1975**, *122* (5), 711.
(4) Peter, L. Kinetics and mechanisms of light-driven reactions at semiconductor electrodes: Principles and techniques. In *Photoelectrochemical Water Splitting: Materials, Processes and Architectures*, Lewerenz, H.-J., Peter, L. Eds.; The Royal Society of Chemistry, 2013; pp 19-51.
(5) Kennedy, J. H.; Frese, K. W. Flatband Potentials and Donor Densities of Polycrystalline α - Fe_2O_3 Determined from Mott-Schottky Plots. *Journal of The Electrochemical Society* **1978**, *125* (5), 723-726.
(6) Tomkiewicz, M. The Potential Distribution at the TiO_2 Aqueous Electrolyte Interface. *Journal of The Electrochemical Society* **1979**, *126* (9), 1505-1510.
(7) Cooper, G.; Turner, J. A.; Nozik, A. J. Mott-Schottky Plots and Flatband Potentials for Single Crystal Rutile Electrodes. *Journal of The Electrochemical Society* **1982**, *129* (9), 1973-1977.
(8) Bian, H.-t.; Guo, Y.; Wang, H.-f. Non-parabolic potential dependence of optical second harmonic generation from the Si(111) electrode/electrolyte interface. *Physical Chemistry Chemical Physics* **2018**, *20* (46), 29539-29548.
(9) Pu, P.; Cachet, H.; Sutter, E. M. M. Electrochemical impedance spectroscopy to study photo-induced effects on self-organized TiO_2 nanotube arrays. *Electrochimica Acta* **2010**, *55* (20), 5938-5946.
(10) Kobayashi, H.; Mizuno, F.; Nakato, Y. Improvement in Hydrogen Photoevolution Efficiency for Platinum-Deposited Indium Phosphide Electrodes by the Removal of Surface States. *Japanese Journal of Applied Physics* **1994**, *33* (Part 1, No. 10), 6065-6070.
(11) Chandrasekaran, K.; Bockris, J. O. M. Determination of concentration of surface states at the illuminated semiconductor—electrolyte interface. *Electrochimica Acta* **1987**, *32* (9), 1393-1402.
(12) Dare-Edwards, M. P.; Hamnett, A.; Trevellick, P. R. Alternating-current techniques in semiconductor electrochemistry. *Journal of the Chemical Society, Faraday Transactions 1: Physical Chemistry in Condensed Phases* **1983**, *79* (9), 2111.
(13) Memming, R. Solid–Liquid Interface. In *Semiconductor Electrochemistry*, 2015; pp 89-125.
(14) Bard, A. J.; Bocarsly, A. B.; Fan, F. R. F.; Walton, E. G.; Wrighton, M. S. The concept of Fermi level pinning at semiconductor/liquid junctions. Consequences for energy conversion efficiency and selection of useful solution redox couples in solar devices. *Journal of the American Chemical Society* **1980**, *102* (11), 3671-3677.
(15) Tomkiewicz, M. The Nature of Surface States on Chemically Modified TiO_2 Electrodes. *Journal of The Electrochemical Society* **1980**, *127* (7), 1518-1525.
(16) Kong, D.-S.; Wei, Y.-J.; Li, X.-X.; Zhang, Y.; Feng, Y.-Y.; Li, W.-J. pH Dependent Behavior and Effects of Photoinduced Surface States during Water Photooxidation at TiO_2/Solution Interface: Studied by Capacitance Measurements. *Journal of The Electrochemical Society* **2014**, *161* (3), H144-H153.
(17) Zhang, Y.; Zhang, H.; Liu, A.; Chen, C.; Song, W.; Zhao, J. Rate-Limiting O–O Bond Formation Pathways for Water Oxidation on Hematite Photoanode. *Journal of the American Chemical Society* **2018**, *140* (9), 3264-3269.
(18) Ma, Y.; Pendlebury, S. R.; Reynal, A.; Le Formal, F.; Durrant, J. R. Dynamics of photogenerated holes in undoped $BiVO_4$ photoanodes for solar water oxidation. *Chemical Science* **2014**, *5* (8), 2964-2973.
(19) Klahr, B.; Gimenez, S.; Fabregat-Santiago, F.; Hamann, T.; Bisquert, J. Water Oxidation at Hematite Photoelectrodes: The Role of Surface States. *Journal of the American Chemical Society* **2012**, *134* (9), 4294-4302.
(20) Kelly, J. J.; Memming, R. The Influence of Surface Recombination and Trapping on the Cathodic Photocurrent at p-Type III-V Electrodes. *Journal of The Electrochemical Society* **1982**, *129* (4), 730-738.

(21) Van Den Meerakker, J. E. A. M. The reduction of iodine at GaAs: the role of potential-redistribution at the semi-conductor/electrolyte interface. *Electrochimica Acta* **1985**, *30* (4), 435-440.
(22) van den Meerakker, J. E. A. M.; Kelly, J. J.; Notten, P. H. L. The Minority Carrier Recombination Resistance: A Useful Concept in Semiconductor Electrochemistry. *Journal of The Electrochemical Society* **1985**, *132* (3), 638-642.
(23) Lincot, D.; Vedel, J. Recombination and charge transfer at the illuminated n-CdTe/electrolyte interface: Simplified kinetic model. *Journal of Electroanalytical Chemistry and Interfacial Electrochemistry* **1987**, *220* (2), 179-200.
(24) Meissner, D.; Lauermann, I.; Memming, R.; Kastening, B. Photoelectrochemistry of cadmium sulfide. 2. Influence of surface-state charging. *The Journal of Physical Chemistry* **1988**, *92* (12), 3484-3488.
(25) Meissner, D.; Memming, R.; Kastening, B. Photoelectrochemistry of cadmium sulfide. 1. Reanalysis of photocorrosion and flat-band potential. *The Journal of Physical Chemistry* **1988**, *92* (12), 3476-3483.
(26) Iqbal, A.; Hossain, M. S.; Bevan, K. H. The role of relative rate constants in determining surface state phenomena at semiconductor–liquid interfaces. *Physical Chemistry Chemical Physics* **2016**, *18* (42), 29466-29477.
(27) Kaufman, A. J.; Nielander, A. C.; Meyer, G. J.; Maldonado, S.; Ardo, S.; Boettcher, S. W. Absolute band-edge energies are over-emphasized in the design of photoelectrochemical materials. *Nature Catalysis* **2024**, *7* (6), 615-623.
(28) Nakato, Y.; Tsumura, A.; Tsubomura, H. Photo- and electroluminescence spectra from an n-titanium dioxide semiconductor electrode as related to the intermediates of the photooxidation reaction of water. *The Journal of Physical Chemistry* **1983**, *87* (13), 2402-2405.
(29) Wilson, R. H. Observation and Analysis of Surface States on TiO_2 Electrodes in Aqueous Electrolytes. *Journal of The Electrochemical Society* **1980**, *127* (1), 228-234.
(30) Salvador, P. Mechanisms of water photooxidation at n-TiO_2 rutile single crystal oriented electrodes under UV illumination in competition with photocorrosion. *Progress in Surface Science* **2011**, *86* (1-2), 41-58.
(31) Tafalla, D.; Salvador, P. Analysis of the photocurrent transient behaviour associated with flatband potential shifts during water splitting at n-TiO_2 electrodes. *Journal of Electroanalytical Chemistry and Interfacial Electrochemistry* **1989**, *270* (1), 285-295.
(32) Tafalla, D.; Pujadas, M.; Salvador, P. Direct measurements of flat-band potential shifts under illumination of the semiconductor-electrolyte interface by electrolyte electroreflectance. *Surface Science* **1989**, *215* (1), 190-200.
(33) Salvador, P. Subbandgap photoresponse of n-TiO_2 electrodes: Transient photocurrent-time behaviour. *Surface Science* **1987**, *192* (1), 36-46.
(34) Salvador, P. Kinetic approach to the photocurrent transients in water photoelectrolysis at n-titanium dioxide electrodes. 1. Analysis of the ratio of the instantaneous to steady-state photocurrent. *The Journal of Physical Chemistry* **1985**, *89* (18), 3863-3869.
(35) Nakamura, R.; Nakato, Y. Primary Intermediates of Oxygen Photoevolution Reaction on TiO_2 (Rutile) Particles, Revealed by *in Situ* FTIR Absorption and Photoluminescence Measurements. *Journal of the American Chemical Society* **2004**, *126* (4), 1290-1298.
(36) Valdés, Á.; Qu, Z. W.; Kroes, G. J.; Rossmeisl, J.; Nørskov, J. K. Oxidation and Photo-Oxidation of Water on TiO_2 Surface. *The Journal of Physical Chemistry C* **2008**, *112* (26), 9872-9879.
(37) Kafizas, A.; Ma, Y.; Pastor, E.; Pendlebury, S. R.; Mesa, C.; Francàs, L.; Le Formal, F.; Noor, N.; Ling, M.; Sotelo-Vazquez, C.; Carmalt, C. J.; Parkin, I. P.; Durrant, J. R. Water Oxidation Kinetics of Accumulated Holes on the Surface of a TiO_2 Photoanode: A Rate Law Analysis. *ACS Catalysis* **2017**, *7* (7), 4896-4903.
(38) Imanishi, A.; Okamura, T.; Ohashi, N.; Nakamura, R.; Nakato, Y. Mechanism of Water Photooxidation Reaction at Atomically Flat TiO_2 (Rutile) (110) and (100) Surfaces: Dependence on Solution pH. *Journal of the American Chemical Society* **2007**, *129* (37), 11569-11578.
(39) Le Formal, F.; Sivula, K.; Grätzel, M. The Transient Photocurrent and Photovoltage Behavior of a Hematite Photoanode under Working Conditions and the Influence of Surface Treatments. *The Journal of Physical Chemistry C* **2012**, *116* (51), 26707-26720.
